# Strong Oxide‐Support Interaction over IrO_2_/V_2_O_5_ for Efficient pH‐Universal Water Splitting

**DOI:** 10.1002/advs.202104636

**Published:** 2022-02-12

**Authors:** Xiaozhong Zheng, Minkai Qin, Shuangxiu Ma, Yuzhuo Chen, Honghui Ning, Rui Yang, Shanjun Mao, Yong Wang

**Affiliations:** ^1^ Advanced Materials and Catalysis Group State Key Laboratory of Clean Energy Utilization Center of Chemistry for Frontier Technologies Institute of Catalysis Department of Chemistry Zhejiang University Hangzhou 310028 P. R. China; ^2^ College of Chemistry and Molecular Engineering Zhengzhou University Zhengzhou 450001 China

**Keywords:** oxides, oxygen evolution reaction, pH‐universal electrocatalysts, strong oxides‐support interaction, water splitting

## Abstract

Constructing strong oxide‐support interaction (SOSI) is compelling for modulating the atomic configurations and electronic structures of supported catalysts. Herein, ultrafine iridium oxide nanoclusters (≈1 nm) are anchored on vanadium oxide support (IrO_2_/V_2_O_5_) via SOSI. The as made catalyst, with a unique distorted IrO_2_ structure, is discovered to significantly boost the performance for pH‐universal oxygen evolution reaction (OER). Based on experimental results and theoretical calculations, the distorted IrO_2_ active sites with flexible redox states in IrO_2_/V_2_O_5_ server as electrophilic centers balance the adsorption of oxo‐intermediates and effectively facilitate the process of O—O coupling, eventually propelling the fast turnover of water oxidation. As a result, IrO_2_/V_2_O_5_ demonstrates not only ultralow overpotentials at 10 mA cm^−2^ (266 mV, pH = 0; 329 mV, pH = 7; 283 mV, pH = 14) for OER, but also high‐performance overall water electrolysis over a broad pH range, with a potential of mere 1.50 V (pH = 0), 1.65 V (pH = 7) or 1.49 V (pH = 14) at 10 mA cm^−2^. In addition, SOSI can simultaneously secure the distorted active sites and thus remarkably improving the catalytic stability, making it a promising strategy to develop high‐performance catalytic systems.

## Graphical abstract

Here, highly dispersed IrO_2_/V_2_O_5_ catalyst is successfully manufactured via strong oxide‐support interaction (SOSI), the distorted IrO_2_ active sites with flexible redox states in IrO_2_/V_2_O_5_ server as electrophilic centers to balance the adsorption of oxo‐intermediates and effectively propel the fast turnover of water oxidation, eventually achieving high‐performance water splitting in a wide pH range.

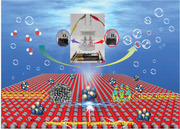

## 1 Introduction

Electricity‐driven water splitting to produce hydrogen (H_2_) powered by renewable energy has been considered a prospective strategy to develop sustainable, fossil‐free and global‐scale energy systems.^[^ ^1^, ^2^, ^3^ ^]^ Electrocatalysts are the central part of the hydrogen and oxygen evolution reactions (HER and OER). Significant success has been realized in the field of HER and overpotential at 10 mA cm^−2^ can be lower than 20 mV.^[^ ^4^, ^5^, ^6^ ^]^ To date, the large‐scale industrial water electrolysis is hindered by the huge energy consumption derived from the large overpotential of OER.^[^ ^7^, ^8^, ^9^, ^10^ ^]^ Basically, producing one oxygen molecule involves the sluggish kinetics of four electrons transfer and the formation of O—O bond.^[^ ^9^, ^10^ ^]^ Consequently, exploring advanced OER electrocatalysts is vital for improving the energy conversion efficiency of water electrolyzers and has been drawn considerable attention in recent years.^[^ ^11^, ^12^, ^13^ ^]^ In principle, the binding energies of multiple intermediates (*OH, *O, and*OOH) in OER are correlated with each other. Typically, the adsorption energy difference between *OOH and *OH is about 3.2 ± 0.2 eV because of scaling relation of the two intermediates without regarding of catalyst type.^[^ ^14^, ^15^, ^16^ ^]^ According to the volcano plot of *η*_OER_ versus (Δ*G*_O*_ − Δ*G*_OH*_), the catalyst with oxygen binding that is neither too strong nor too weak shows optimal activity.^[^ ^14^, ^17^ ^]^ To date, several approaches have been reported for modulating oxygen intermediate adsorption energy, including substituting with foreign elements,^[^ ^18^, ^19^ ^]^ heterostructure modulating,^[^ ^20^, ^21^ ^]^ strain engineering,^[^ ^22^, ^23^ ^]^ nanoscopic confinement^[^ ^24^ ^]^ and so on. So far, the overpotentials values of state‐of‐the‐art OER catalysts at 10 mA cm^−2^ are smaller than 200 mV,^[^ ^25^, ^26^, ^27^ ^]^ however, most of them can only be applied in a certain pH or specific working scenarios. In order to meet the complicate working conditions, it is highly urgent to develop a high‐performance catalyst that is universally compatible to optimize the reliability during operation.

In the past decades, constructing strong metal/metal oxide‐support interaction (SMSI/SOSI) has attracted great attentions due to its intriguing capabilities for designing advanced catalysts with unique properties.^[^ ^28^, ^29^, ^30^, ^31^ ^]^ On the one hand, the electron relocation on the interface will modulate electronic properties of active sites.^[^ ^32^ ^]^ On the other hand, distinctive structure deformation as well as diverse atomic coordination environment would appear at the interface, both of which will significantly affect the electrocatalytic performance.^[^ ^32^ ^]^ Although the idea of manufacturing supported catalysts seems to be clear‐cut, the individual components must be carefully selected for specific purposes to take advantage of this catalyst design strategy. In term of constructing supported OER electrocatalysts, IrO_2_ and VO*_x_* (1 ≤ *x* ≤ 2.5) would be appropriate building blocks. Though IrO_2_ is an excellent benchmark catalyst for OER in acidic media, the binding between IrO_2_ and oxygen‐related intermediates is still strong, limiting its OER performance.^[^ ^14^, ^33^ ^]^ A large space exists for the property enhancement of IrO_2_ by modifying geometric and electronic structures, which can lead to optimized interaction between the catalyst and oxygen intermediates.^[^ ^34^ ^]^ Recently, owing to their excellent interactions with molecules or ions, outstanding catalytic activities, and/or strong electron‐electron correlations, nanostructured vanadium oxides‐based composite materials have been extensively studied for energy conversion (water splitting, carbon dioxide reduction and alcohol oxidation reactions) in the recent years.^[^ ^35^, ^36^, ^37^, ^38^, ^39^ ^]^ Benefit from the multiple valence states, V_2_O_5_ exhibits redox and Lewis acid/base properties, which makes strong interaction with foreign active materials. On the one hand, strong interaction is beneficial to regulate the intrinsic activity of active species, and on the other hand, it also plays a role in stabilizing the substrate. Additionally, vanadium oxide can facilitate the hydrolysis kinetics and provide hydrogen protons during electrochemical process, which has been considered important in surface catalytic reactions.^[^ ^40^ ^]^ Inspired by the aforementioned discussions, we envisioned whether a rational modification of IrO_2_ on VO*_x_* support could integrally construct a supported catalyst with strong oxide‐support interaction, and improve catalytic activity and durability.

Herein, a simple method was developed to prepare highly dispersed IrO_2_/V_2_O_5_ catalysts in which vanadium containing MOF MIL‐88B (V) was used as a host. After impregnating with Ir precursor and then annealing under air atmosphere, IrO_2_ nanoclusters (≈1 nm), with unique distorted and deformed lattice fringes induced by SOSI, are uniformly embedded in the porous network of V_2_O_5_. Contrast experiments and theoretical calculations indicated that the SOSI between IrO_2_ and V_2_O_5_, i.e., the SOSI induced lattice fringes’ distortion exposed more catalytic active sites and enhanced the redox states of electrophilic IrO_2_, can significantly promote the OER performance of IrO_2_ over a wide pH range (pH = 0–14, 1–2 orders of magnitude higher intrinsic and mass activity than commercial IrO_2_) as well as durability.

## 2 Results and Discussion

### 2.1 Preparation and Structure Characterization of Catalysts

**Scheme 1:**
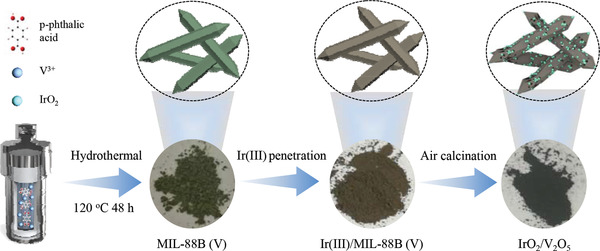
Schematic illustration of the preparation of IrO_2_/V_2_O_5_ hybrid oxides electrocatalysts.

**Figure 1:**
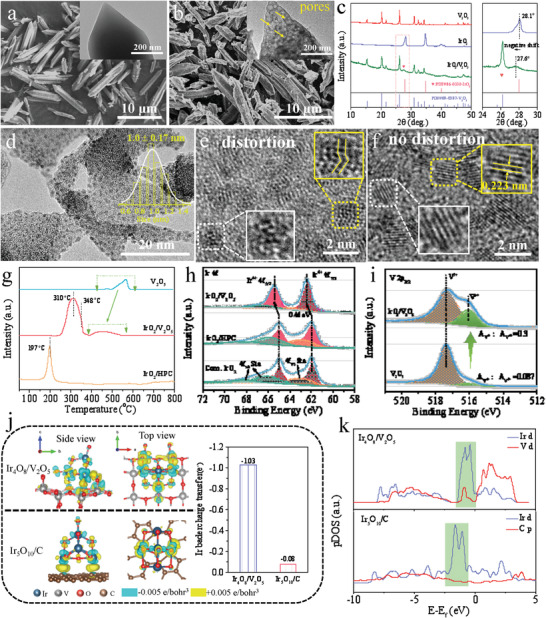
Morphology and structural characteristics of catalysts. The SEM images of a) MIL‐88B (V) and b) IrO_2_/V_2_O_5_; the inserts show corresponding TEM images. c) XRD patterns of IrO_2_/V_2_O_5_, V_2_O_5_, and IrO_2_. d,e) The HRTEM images of IrO_2_/V_2_O_5_; the insert in (d) is the size distribution pattern. f) The HRTEM image of IrO_2_/HPC; g) H_2_‐TPR patterns of IrO_2_/V_2_O_5_, IrO_2_/HPC, and V_2_O_5_. h) Ir 4f spectra of IrO_2_/V_2_O_5_, IrO_2_/HPC, and commercial IrO_2_; i) V 2p_3/2_ spectra of IrO_2_/V_2_O_5_ and V_2_O_5_. j) Top and side views of charge density difference in the interface of Ir_4_O_8_/V_2_O_5_ and Ir_5_O_10_/C (the cyan region reflects an electron‐deficient state while the yellow region reflects an electron‐rich area); the insert shows Ir Bader charge transfer of Ir_4_O_8_/V_2_O_5_ and Ir_5_O_10_/C. k) The pDOS of d orbitals of Ir and V in Ir_4_O_8_/V_2_O_5_, and the d orbitals of Ir and p orbitals of C in Ir_5_O_10_/C, respectively.

The synthesis of the IrO_2_/V_2_O_5_ hybrid oxides is illustrated in **Scheme** **1**, which includes the synthesis of the Ir(III)/MIL‐88B (V) precursors and the subsequent thermal pyrolysis. First of all, MIL‐88B (V) was synthesized by scalable hydrothermal method,^[^ ^41^ ^]^ in which vanadium chloride and p‐phthalic acid were used as raw materials. And then Ir(III) ions can be readily hosted within MIL‐88B (V) by means of impregnation. An apparent color changes from dark green to brown can be observed over the parent MIL‐88B (V) and Ir(III)/MIL‐88B (V), which indicates the successful incorporation of the Ir(III) ions into MIL‐88B (V). The power X‐ray diffractions (XRD) patterns of both MIL‐88B (V) and Ir(III)/MIL‐88B (V) (Figure S1, Supporting Information) show the similar characteristic peaks, suggesting that the presence of Ir(III) (Figure S2, Supporting Information) has no apparent influence on the crystalline structure of MIL‐88B (V). Meanwhile, there was no peak for IrCl_3_ or other Ir‐V mixed materials, indicating that IrCl_3_ did not crystalize or interact with MIL‐88B (V) to form Ir‐V mixed material in the pores of MIL‐88B (V) but was adsorbed on the pore surface. Scanning electron microscopy (SEM) and transmission electron microscopy (TEM) images of MIL‐88B (V) and Ir‐MIL‐88B (V) exhibit similar shuttle‐like morphology (**Figure** **1**a and Figure S3, Supporting Information), and element distribution analysis of Ir‐MIL‐88B (V) indicates the Ir and V atoms are uniformly distributed to form the hybrids. Subsequently, black powder IrO_2_/V_2_O_5_ was obtained by annealing Ir(III)/MIL‐88B (V) under air atmosphere. As a comparison, MIL‐88B (V) was treated in the same condition to produce yellow powder V_2_O_5_. As shown in Figure 1b, IrO_2_/V_2_O_5_ inherits the original shuttle‐like morphology of MIL‐88B (V) with rough surface and porous structure. However, SEM and TEM images (Figure S4, Supporting Information) of V_2_O_5_ exhibit rod‐like morphology composited of many nanoplates, affirming that introduction of Ir is beneficial to stabilize the shuttle‐like structure and form porous skeleton during postannealing process. Nitrogen adsorption/desorption measurement evidenced that the specific surface area of IrO_2_/V_2_O_5_ is 40 m^2^ g^−1^, which is higher than that of V_2_O_5_ (18 m^2^ g^−1^) as shown in Figure S5 (Supporting Information). XRD measurement (Figure 1c) demonstrates typical tetragonal IrO_2_ (JCPDS No. 86–0330) and orthorhombic V_2_O_5_ (JCPDS No. 09–0387) phase in IrO_2_/V_2_O_5_. Compared to IrO_2_ analog, however, the (110) characteristic reflection in the IrO_2_/V_2_O_5_ sample broadens and shifts to a lower degree at 2*θ* = 27.6°, indicating an lattice expansion feature of IrO_2_ induced by the known effects of strain on both the position and width of diffraction peaks.^[^ ^42^, ^43^ ^]^ Moreover, high‐resolution transmission electron microscope (HRTEM) image and corresponding line‐scan profile (Figure 1d and Figures S6 and S7, Supporting Information) demonstrate that IrO_2_ nanoclusters (NCs) with mean size of ≈1 nm are uniformly embedded on porous skeleton of V_2_O_5_. Scanning transmission electron microscopy (STEM) and corresponding elemental mapping images (Figure S8, Supporting Information) elucidate that Ir, V, and O elements are uniformly distributed over the region, and Ir loading is 20.8 wt%, which is consistent with the result of inductively coupled plasma‐optical emission spectrometry (ICP‐OES，19.7 wt%) (Table S1, Supporting Information). Further investigating the IrO_2_ nanoclusters, the distorted and deformed lattice fringes of IrO_2_ are observed in the HRTEM image (Figure 1e), corroborating the XRD results, indicating a possible SOSI between IrO_2_ and V_2_O_5_. To systematically investigate the possible SOSI, we delicately designed hierarchically porous carbon^[^ ^44^ ^]^ supported IrO_2_ catalysts (IrO_2_/HPC) with similar particle size distribution and Ir loading (19.1 wt%) of IrO_2_ (TableS1 and Figure S9, Supporting Information). As shown in Figure 1f, IrO_2_/HPC sample possesses regular lattice fringes (interplanar distances of 0.223 nm) without obvious distortion. Furthermore, IrO_2_ was loaded on the synthesized V_2_O_5_ by wet impregnation and postcalcination as comparison. Although, it is not as obvious as the IrO_2_/V_2_O_5_ sample, HRTEM images and XRD pattern indeed showed the formation of distorted IrO_2_ NCs (Figure S10, Supporting Information). These characterizations unambiguously unveiled that the SOSI exists between IrO_2_ NCs and V_2_O_5_ support.

### 2.2 In‐Depth Understanding of SOSI Effect of IrO_2_/V_2_O_5_

To further figure out the effect of SOSI on the properties of catalysts, H_2_ temperature programmed reduction (H_2_‐TPR), X‐ray photoelectron spectroscopy (XPS), and density functional theory (DFT) calculations were carried out. Figure 1g shows the H_2_‐TPR profiles of IrO_2_/V_2_O_5_, IrO_2_/HPC and V_2_O_5_. The reduction profile of IrO_2_/HPC is characterized by a single and sharp peak at 197 °C, assigned to the reduction of iridium oxide species,^[^ ^45^, ^46^ ^]^ which is similar to reduction temperature of commercial IrO_2_ (210 °C) (Figure S11, Supporting Information). Pure V_2_O_5_ material presents main hydrogen consumption peaks at about 430–590 °C, showing high oxidation state (V) of V reduced to low oxidation state.^[^ ^47^ ^]^ With regard to IrO_2_/V_2_O_5_ hybrid oxide, the H_2_‐TPR profile shows the typically two reduction peaks. Obvious shift and broaden of iridium oxide reduction peak to a higher temperature region (310 °C) is clearly observed, indicating that the iridium oxide species supported on V_2_O_5_ are more difficult to be reduced. What's more, the shoulder peak around 348 °C is detected, which may be attributed to iridium oxide species intimately interacted with the V_2_O_5_ support at the interface. Meanwhile, the reduction temperature of V_2_O_5_ species in IrO_2_/V_2_O_5_ moves to lower temperature region (440–550 °C) comparison to pure V_2_O_5_. Based on the results of H_2_‐TPR measurement, we convinced that the strong interaction between IrO_2_ species and V_2_O_5_ support is successfully constructed.

XPS was carried out to characterize the surface structure of the catalyst. As shown in Figure 1h,i and Figure S12 (Supporting Information), in the typical Ir 4f spectra of commercial IrO_2_ and IrO_2_/HPC (fit parameters are provided in Table S2, Supporting Information), the peaks at around 61.95 and 64.95 eV can be mainly attributed to 4f_7/2_ and 4f_5/2_ of Ir^4+^, respectively, followed by three satellite peaks.^[^ ^24^, ^48^ ^]^ Compared with commercial IrO_2_ and IrO_2_/HPC, it is clear that the binding energy of Ir^4+^ for IrO_2_/V_2_O_5_ is positively shifted by 0.46 eV up to 62.41 and 65.41 eV. Combining with the discussion of H_2_‐TPR, we claimed that a decreased charge density of Ir is responsible for the higher binding energy rather than forming Ir^3+^ with a reverse core level shift. In the V 2p_3/2_ XPS spectra (details see Table S3, Supporting Information), the V^5+^ peak at 517.30 eV is dominant in the pure V_2_O_5_ sample, accompanied by weak peak of V^4+^ centered at 516.05 eV (A_V_^4+^:A_V_^5+^ = 0.087). As excepted, apart from typical V^5+^ signal, the intensity of V^4+^ peak significantly enhances in IrO_2_/V_2_O_5_ hybrid oxide (A_V_^4+^:A_V_^5+^ = 0.3).^[^ ^49^ ^]^ These results point to the highly strong electron coupling effect between IrO_2_ NCs and V_2_O_5_ support with the charge transfer from the IrO_2_ to V_2_O_5_. DFT calculations were additionally performed for analyzing the charge transfer and internal interactions between IrO_2_ NCs and V_2_O_5_ and C supports, respectively. As shown in Figure 1j, we obviously observe that the strong charge redistribution occurs at the interface region of IrO_2_/V_2_O_5_. According to Bader charge analysis, a partial charge transfer of 1.03 e^−^ from Ir to the V_2_O_5_ was found, while only charge transfer of 0.08 e^−^ is discovered in IrO_2_/C system, which is in good agreement with the XPS analysis. The projected density of states (pDOS) curves of IrO_2_/V_2_O_5_ (Figure 1k) show that the peaks of Ir d orbitals and V d orbitals overlap well around Femi level, affirming the strong interaction between IrO_2_ and V_2_O_5_. In contrast, the pDOS curves of IrO_2_/C elucidate that less orbitals coupling between Ir d orbitals and C p orbitals leads to the weak interaction.

### 2.3 Catalytic Activity toward OER

**Figure 2:**
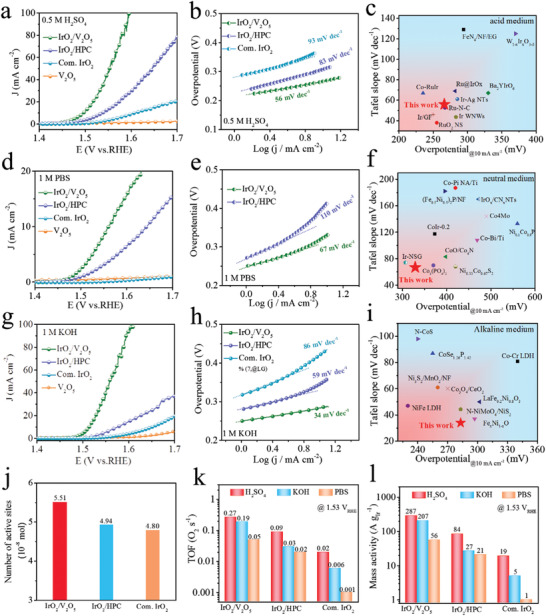
OER activity comparison (Ir loading with 0.1 mg cm^−2^). a) The steady‐state polarization curves were obtained with a low scan rate of 1 mV s^−1^. b) Tafel plots of different electrocatalysts in 0.5 м H_2_SO_4_, and c) comparison of acidic OER activity between IrO_2_/V_2_O_5_ and recently reported electrocatalysts. d) The steady‐state polarization curves. e) Tafel plots of different electrocatalysts in 1 м PBS, and f) comparison of neutral OER activity between IrO_2_/V_2_O_5_ and recently reported electrocatalysts. g) The steady‐state polarization curves, h) Tafel plots of different electrocatalysts in 1 м KOH, and i) comparison of alkaline OER activity between IrO_2_/V_2_O_5_ and recently reported electrocatalysts. j) The number of active sites in IrO_2_/V_2_O_5_, IrO_2_/HPC, and commercial IrO_2_ measured with Cu‐UPD method. k) TOF values and l) mass activity of IrO_2_/V_2_O_5_, IrO_2_/HPC, and commercial IrO_2_ at 1.53 V_RHE_ for OER.

The catalytic properties of IrO_2_/V_2_O_5_ and IrO_2_/HPC toward the OER were then systematically evaluated in a typical three‐electrode set‐up in a wide pH range (0–14), along with commercial rutile IrO_2_ (consisted of 20–300 nm irregular particles, see Figure S13, Supporting Information) and V_2_O_5_ substrate as comparison. Prior to this, by optimizing the synthesis conditions of IrO_2_/V_2_O_5_, 400 °C and 4 h are suitable calcination temperature and time to obtain optimal OER performance for subsequent comparison (Figures S14–S16, Supporting Information). Specially, IrO_2_/V_2_O_5_ gives an extremely low OER overpotential of 266 mV at 10 mA cm^−2^ (**Figure** **2**a,b) and a Tafel slope of 56 mV dec^−1^ in and 0.5 м H_2_SO_4_, both significantly lower than that of IrO_2_/HPC (307 mV, 83 mV dec^−1^), commercial IrO_2_ (379 mV, 93 mV dec^−1^), and V_2_O_5_ support. Notably, the acidic OER catalytic activity is also superior to most of the recently reported landmark catalysts (Figure 2c and Table S4, Supporting Information). When electrolyte is switched to 1 м PBS (Figure 2d,e), as expected, IrO_2_/HPC and commercial IrO_2_ display almost inert or inferior performance as result of intrinsic sluggish kinetics under neutral condition. In stark contrast, IrO_2_/V_2_O_5_ still demonstrates unprecedented catalytic capability with a small overpotential of 329 mV at 10 mA cm^−2^. Tafel slope plots further elucidate the accelerated kinetics of IrO_2_/V_2_O_5_ (67 mV dec^−1^) in neutral‐pH media, which opens the door to the realization of efficient and biocompatible systems for energy storage and conversion, as well as for direct seawater splitting.^[^ ^50^, ^51^ ^]^ The comparison of OER activity with state‐of‐art catalysts also indicates that IrO_2_/V_2_O_5_ ranks among the top neutral OER catalysts (Figure 2f and Table S5, Supporting Information). Undoubtedly, IrO_2_/V_2_O_5_ also performs well in 1 м KOH electrolyte, requiring a low overpotential of 283 mV to attain 10 mA cm^−2^ and an unprecedented low Tafel slope of 34 mV dec^−1^, far superior to those of IrO_2_/HPC, commercial IrO_2_ and outstanding reported alkaline OER catalysts (Figure 2g–i and Table S6, Supporting Information). It is worth mentioning that electrocatalytic activity is tightly connected with the capability of charge transfer, the number of active sites and turnover rates.^[^ ^52^, ^53^, ^54^, ^55^ ^]^ Therefore, the electrochemical impedance spectroscopy (EIS) was first performed to investigate the interface charge transfer process of the electrode. The semicircular diameter of IrO_2_/V_2_O_5_ in Nyquist plots is much smaller than that of IrO_2_/HPC, commercial IrO_2_ and V_2_O_5_ in all‐pH range, as revealed in Figure S17 (Supporting Information), which indicates smaller charge transfer resistance induced by rapid interfacial electron migration between distorted IrO_2_ and V_2_O_5_ support. The high density of active sites plays an important role in the improvement of catalytic activity. In order to quantitatively analyze the number of active sites, underpotential deposition of copper (Cu‐UPD) was then conducted to measure the active site density.^[^ ^55^ ^]^ As is seen in Figure 2j and Figure S18 (Supporting Information), compared with commercial IrO_2_ (4.80 × 10^−8 ^mol) and IrO_2_/HPC (4.94 × 10^−8^ mol), an increase in the number of active sites can be observed for IrO_2_/V_2_O_5_ (5.51 × 10^−8^ mol). As mentioned above, the small NCs (≈1 nm) consists of unique structure distortion, which endows the IrO_2_/V_2_O_5_ catalyst with a high exposure of IrO_2_ active sites as well as a high accessibility for substrate molecules. The turnover frequencies (TOFs) based on the number of active sites of the catalyst was calculated to evaluate the intrinsic activity of a catalyst. As illustrated in Figure 2k and Figure S19 (Supporting Information), with the overpotential increase to 300 mV of OER, the TOF values of IrO_2_/V_2_O_5_ even reach 0.27, 0.19, and 0.05 O_2_ s^−1^, which are about 3.0, 6.3, and 2.5 folds of those of IrO_2_/HPC (0.09, 0.03, and 0.02 O_2_ s^−1^), and far surpassing commercial IrO_2_ in acidic, alkaline and neutral media, respectively. As one of precious metal, the mass activity of Ir‐based electrocatalysts is of great importance for industrial application. Mass activity of IrO_2_/V_2_O_5_ is 287, 207, and 56 A g_Ir_^−1^, which is about 3.4, 7.7, and 2.7 folds of those of IrO_2_/HPC (84, 27, and 21 A g_Ir_^−1^), and 1–2 orders of magnitude larger than those of commercial IrO_2_ in acidic, alkaline and neutral media at the overpotential of 300 mV (Figure 2l and Figure S20, Supporting Information), respectively, strongly verifying the enhanced intrinsic activity of IrO_2_/V_2_O_5_.

**Figure 3:**
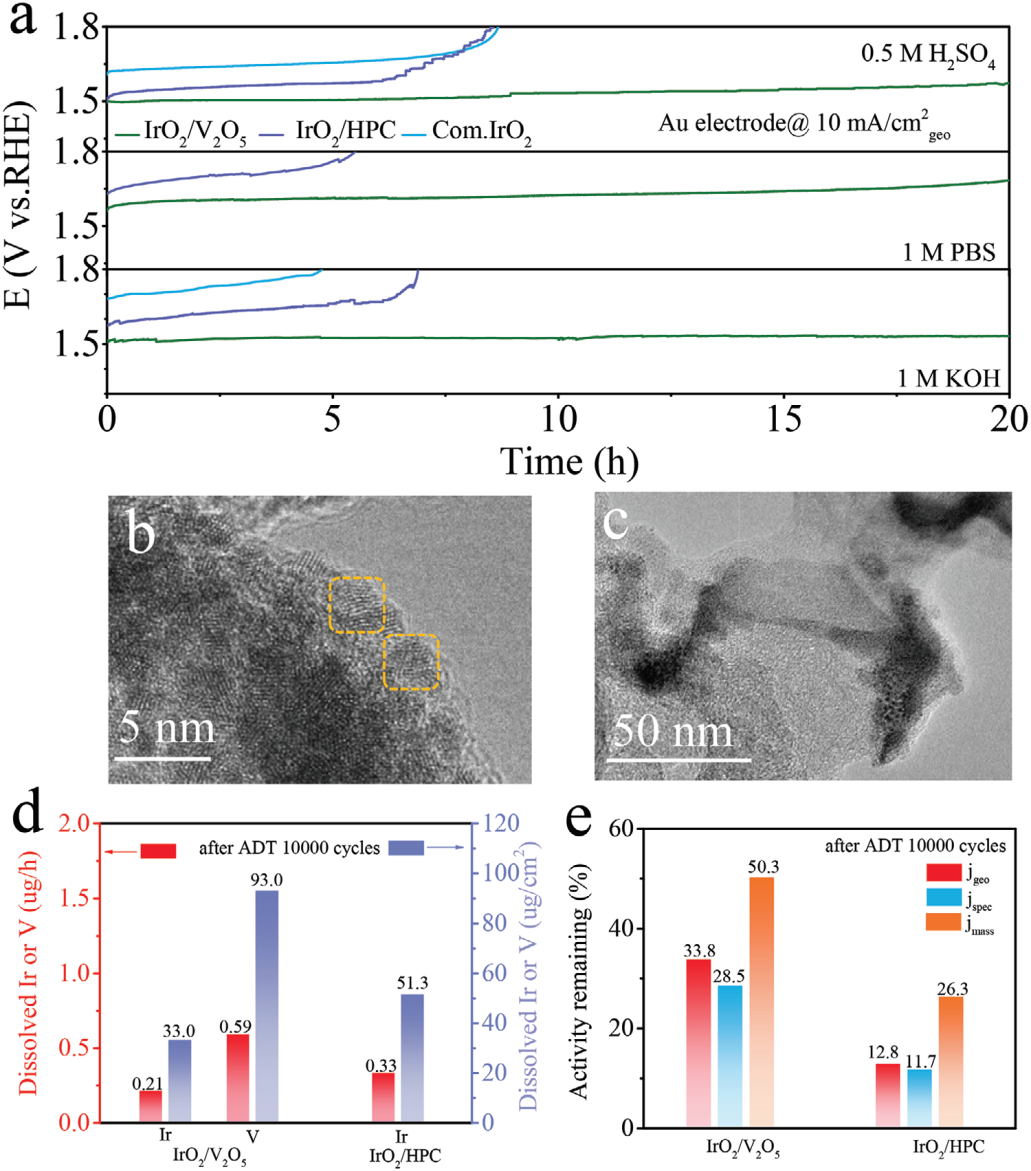
The stability tests of catalysts. a) Chronopotentiometry tests of IrO_2_/V_2_O_5_, IrO_2_/HPC, and commercial IrO_2_ for OER at 10 mA cm^−2^ current density on Au electrode in 0.5 м H_2_SO_4_, 1 м PBS, and 1 м KOH. b,c) The typically TEM images of IrO_2_/V_2_O_5_ and IrO_2_/HPC after long‐time stability test and the mark in (b) shows the distorted IrO_2_ cluster of IrO_2_/V_2_O_5_. d) The dissolved Ir or V content normalized to time and to the geometric surface area after ADT 10 000 cycles in 0.5 м H_2_SO_4_. e) Activity remaining (*j*_geo_, *j*_spec_, and *j*_mass(Ir)_) of the ADT samples at 1.53 V_RHE_.

Stability is an important criterion to evaluate the performance of catalysts. The stability of the IrO_2_/V_2_O_5_, IrO_2_/HPC and commercial IrO_2_ catalysts were assessed by by *static* chronopotentiometry test and *transient* accelerated degradation test (ADT) protocol on Au electrode (avoiding the influence of back electrode to stability analysis).^[^ ^56^, ^57^ ^]^ The *static* chronopotentiometry test (at 10 mA cm^−2^) is conventional way to evaluate the stability, as plotted in **Figure** **3**a, the IrO_2_/V_2_O_5_ catalyst represents a relatively stable horizontal line in different electrolyte (even in the acidic media) during 20 operating hours, while IrO_2_/HPC and commercial IrO_2_ exhibit a progressive activity decay within a few hours, demonstrating the outstanding activity retention of IrO_2_/V_2_O_5_, which is competitive to the state‐of‐art Ir‐based OER catalysts reported in literatures (Table S7, Supporting Information). The detailed characterizations of IrO_2_/V_2_O_5_ and IrO_2_/HPC after durability are conducted to reveal the change of structures. The IrO_2_/V_2_O_5_ postcatalyst maintains its original porous skeleton and IrO_2_ nanoclusters are slightly aggregated (Figure 3b and Figure S21, Supporting Information). Although the distorted structure is detected, it is not as obvious as the original state, which is ascribed to the loss of V_2_O_5_ support confirmed by V2p spectra after stability test (Figure S22a, Supporting Information). Ir4f spectra of postcatalyst exhibit the huge change of line‐shape (Figure S22b, Supporting Information), and an amount of Ir(III) species (4f_7/2_: 63.02 eV and 4f_5/2_: 66.02 eV) is available on the surface.^[^ ^58^ ^]^ In sharp contrast, as shown in Figure 3c and Figure S23 (Supporting Information), the IrO_2_ NCs loaded on HPC after stability test are obviously agglomerated. Notably, *static* protocol is not sufficient to evaluate the stability of Ir‐based OER catalysts. Therefore, the *transient* ADT protocol endows the catalyst to distinctly higher currents (double layer charging and fast redox reactions) than the *static* protocol at same potential, which is benefit to save time to investigate the durability. As seen in the Figure S24a–c (Supporting Information), with increasing the numbers of ADTs, the main redox centered at 0.95–1.0 V_RHE_ and shifted to lower potential, which represents the typical transition of Ir^III+^ to Ir^IV+^,^[^ ^50^ ^]^ agreeing with above results of Ir4f spectra. The strong change in redox activity during ADTs is reflected by the increase in *Q*_anodic_ (*Q*_anodic‐final_ = 2.28 mC > *Q*_anodic‐initial =_ 1.92 mC). The same process is performed on IrO_2_/HPC in Figure S25a–c (Supporting Information) for comparison. The leaking extent of Ir or V from the electrode to electrolyte after ADTs test in 0.5 м H_2_SO_4_ are determined by ICP‐MS measurement. To show this more clearly, when the amount of dissolved Ir is normalized to time and to the geometric surface area, as shown in the Figure 3d, the Ir dissolution rate of IrO_2_/V_2_O_5_ (0.21 ug h^−1^ or 33.0 ug cm^−2^) is much smaller than those of IrO_2_/HPC (0.33 ug h^−1^ or 51.3 ug cm^−2^). Meanwhile, we observed that the V/Ir mass ratio of IrO_2_/V_2_O_5_ decreases from initial 2.1 to 1.7 due to the faster V dissolution rate (0.59 ug h^−1^ or 93.0 ug cm^−2^). Furthermore, as displayed in Figure 3e, Figures S24d–f and S25d–f (Supporting Information), the observed activity (*j*_geo_, *j*_spec_, and j_mass_) remaining percentage of IrO_2_/V_2_O_5_ at 1.53 V_RHE_ are 2 times higher than those of IrO_2_/HPC. These results collectively confirm that the SOSI effect between V_2_O_5_ and IrO_2_ can efficiently reduce the detrimental effects of agglomeration of particles and degradation of intrinsic or mass activity of Ir active sites.

### 2.4 DFT Calculations

**Figure 4:**
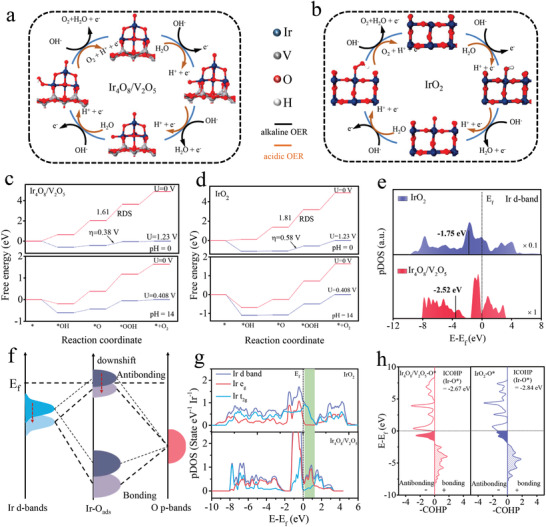
DFT calculations. a,b) Proposed acidic and alkaline OER pathway on Ir_4_O_8_ cluster supported on V_2_O_5_ (001) substrate and rutile IrO_2_ (110) under for DFT calculations. Calculated free energy diagrams of OER on c) Ir_4_O_8_/V_2_O_5_ and d) IrO_2_ under pH = 0 and 14. e) Calculated pDOS of Ir d‐band in Ir_4_O_8_/V_2_O_5_ and IrO_2_. f) Schematic bond formation of oxygen adsorbate on shifting down d‐band centers of Ir. g) Calculated pDOS of Ir active site on Ir_4_O_8_/V_2_O_5_ and IrO_2_; h) –pCOHP curves for O* intermediate adsorption on Ir active site for Ir_4_O_8_/V_2_O_5_ and IrO_2._

DFT calculations were performed to unveil the mechanism of the OER from the view of atomic and electronic structures. Based on the amorphous and distorted iridium oxide structure observed in HRTEM, the OER computational model adopt Ir_4_O_8_ cluster supported on V_2_O_5_ (001) substrate along with periodically interconnected rutile IrO_2_ (110) as comparison. The elementary electrochemical steps for proposed the 4e^−^ mechanism of OER on Ir_4_O_8_/V_2_O_5_ and IrO_2_ under acid and alkaline media are presented in **Figure** **4**a,b, which is following conventional adsorbate evolution mechanism (AEM) pathway.^[^ ^24^, ^59^ ^]^ The Ir atoms in both models serve as the active sites in the OER process. The computed energy landscapes of the OER steps at U = 0 and 1.23 V (acid OER) or 0.408 V (alkaline OER) for Ir_4_O_8_/V_2_O_5_ and IrO_2_ are shown in Figure 4c,d. For both of the calculated configurations, the OER rate‐determining step (RDS) is the formation of Ir‐OOH* intermediate from Ir‐O* with free energy barriers of 0.38 and 0.58 eV on Ir_4_O_8_/V_2_O_5_ and IrO_2_ under the equilibrium potential, respectively, indicating that Ir_4_O_8_/V_2_O_5_ is more referable for OER. As demonstrated in Figure S26 and Table S8 (Supporting Information), compared with IrO_2_ (Δ*E*_O*_ = 1.35 eV, *L*_Ir‐O*_ = 1.78 Å), the obvious reduced oxygen adsorption energy (Δ*E*_O*_ = 2.02 eV) and enlarged Ir—O* bond length (*L* = 1.82 Å) on Ir_4_O_8_/V_2_O_5_ may result in the relatively higher OER activity. This is consistent with the results by Nørskov and coworkers^[^ ^14^ ^]^ that catalyst can bind O too strongly and the overall reaction is limited by the O—O coupling process, and appropriately weakening the strength of M—O* is beneficial to reduce the barrier for the formation of M—OOH*.

Obviously, the SOSI between V_2_O_5_ and IrO_2_ weakens the binding of IrO_2_ with oxygen. To figure out the surface electronic features of catalyst is conducive to better understanding the discrepancy of oxygen adsorption energy, therefore, the d‐band centers of the Ir atoms of Ir_4_O_8_/V_2_O_5_ and IrO_2_ were calculated (Figure 4e). It is found that the d‐band center of Ir atoms (−2.52 eV) of Ir_4_O_8_/V_2_O_5_ is far away from the Fermi level compared to that of IrO_2_ (−1.75 eV). It is well known that the lower the center of d‐band relates, the higher the antibonding states, leading to weak adsorption (Figure 4f). Furthermore, the pDOS curves of Ir d‐bands in IrO_2_/V_2_O_5_ suggest that there are the more unoccupied e_g_ states at 0.2 to 1.2 eV above Fermi level (Figure 4g), which indicated that the V_2_O_5_ support regulates the e_g_ occupancy to affect the bonding of oxygen intermediate in the AEM,^[^ ^12^, ^60^ ^]^ thus promoting OER. To further quantitatively reveal the interaction between Ir active sites and adsorbed O*, crystal orbital Hamilton population (COHP)^[^ ^61^, ^62^ ^]^ were calculated to represent the bonding and antibonding filling states of Ir‐O*. As illustrated in Figure 4h and Figure S27 (Supporting Information), the values of integrated COHP (ICOHP) for Ir—O* bond is computed on Ir_4_O_8_/V_2_O_5_ (−2.67 eV) and IrO_2_ (−2.84 eV) by calculating the energy of COHP integral up to the Fermi level, where the more positive ICOHP value on Ir_4_O_8_/V_2_O_5_ indicates the weaker bonding. In rutile IrO_2_, O—O bond formation may be hindered by its “rigid” periodically linked structure, while distorted structures of Ir_4_O_8_/V_2_O_5_ enables the iridium oxide species more “flexible” during the oxidation‐reduction cycles to accelerate the hydroxyl oxidation and the subsequent O—O bond formation,^[^ ^63^ ^]^ which is supported by the comparison of the cyclic voltammetry (CV) curves of IrO_2_/V_2_O_5_ and IrO_2_ (Figure S28, Supporting Information).

### 2.5 Overall Water Splitting Performance

**Figure 5:**
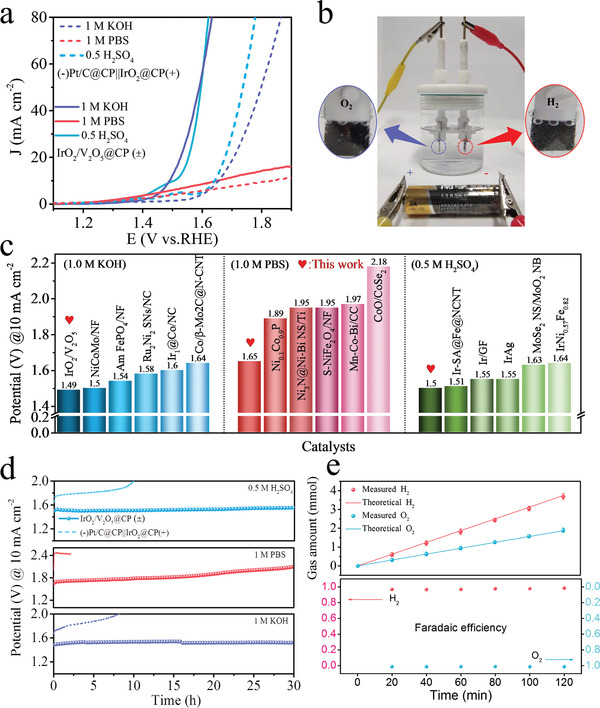
Overall water electrolysis of IrO_2_/V_2_O_5_@CP (±).a) Polarization curves of IrO_2_/V_2_O_5_@CP (±) and commercial (−)Pt/C@CP‐IrO_2_@CP(+) coupled water electrolysis cell in different electrolytes. b) The digital photograph of the water splitting setup with an AA battery with a nominal voltage of 1.5 V. c) Full water splitting potentials of IrO_2_/V_2_O_5_ and reported electrocatalysts for comparison at 10 mA cm^−2^ current density. d) Durability test for the IrO_2_/V_2_O_5_@CP and (−)Pt/C@CP‐IrO_2_@CP(+) samples in a two‐electrode configuration at 10 mA cm^−2^ in different electrolytes. e) The amount of H_2_ and O_2_ theoretically calculated and experimentally measured versus time for IrO_2_/V_2_O_5_@CP (±) at 100 mA cm^−2^ for 120 min as well as corresponding Faradaic efficiency.

Besides, we also evaluated the HER performance of IrO_2_/V_2_O_5_, IrO_2_/HPC, commercial IrO_2_ and V_2_O_5_ in all‐pH media (Figure S29, Supporting Information). Surprisingly, IrO_2_/V_2_O_5_ displays a small overpotential of 65, 176, and 49 mV to achieve 10 mA cm^−2^ in acidic, neutral and alkaline solution, respectively, surpassing those of IrO_2_/HPC (129, 252, and 130 mV) and commercial IrO_2_ (136, 317, and 198 mV). Meanwhile, the IrO_2_/V_2_O_5_ shows lowest Tafel slopes and charge transfer resistance among the catalysts, manifesting its fastest reaction kinetics. Impressively, in all three conditions, IrO_2_/V_2_O_5_ is demonstrated as a very robust electrocatalyst with negligible degradation of activity after 20 h of continuous operation. Hence, the IrO_2_/V_2_O_5_ is expected to act as a “universally compatible” electrocatalyst that simultaneously shows excellent HER and OER performances over the entire pH range. In order to demonstrate the practical applications, we loaded the IrO_2_/V_2_O_5_ electrocatalysts on carbon fiber paper (CP) as anode and cathode to construct a two‐electrode water splitting cell in 0.5 M H_2_SO_4_, 1 m PBS and 1 m KOH solutions (catalyst loading amount is 1 mg cm^−2^). In the experiments of all‐pH overall water splitting, the potential required for two‐electrode IrO_2_/V_2_O_5_@CP (±) electrolyzer is only 1.49, 1.65, and 1.50 V to reach a current density of 10 mA cm^−2^ in alkaline, neutral, and acidic media, exceeding the commercial (−)Pt/C@CP||IrO_2_@CP(+) electrolyzer (**Figure** **5**a). Moreover, a commercial AA battery with a nominal voltage of 1.50 V is able to drive overall water splitting with obvious gas bubble release, confirming the high efficiency of the IrO_2_/V_2_O_5_ catalysts toward both HER and OER (Figure 5b and Movie S1, Supporting Information). These results reveal that the IrO_2_/V_2_O_5_@CP electrode has potential applications in low‐cost and energy‐efficient water electrolysis. Additionally, compared with previously highly active water splitting electrocatalysts, the overall water splitting performance of IrO_2_/V_2_O_5_ catalyst is significantly comparable to the most electrocatalysts in different media (Figure 5c and Table S9, Supporting Information). Besides, IrO_2_/V_2_O_5_@CP (±) system exhibits excellent stability in all‐pH condition by chronopotentiometry tests (Figure 5d), the voltage required for maintaining the overall water splitting current density of 10 mA cm^−2^ remains stable for 30 h. In sharp contrast, (−)Pt/C@CP||IrO_2_@CP(+) electrolyzer deactivates rapidly within a few hours. We detected the generated H_2_ and O_2_ from IrO_2_/V_2_O_5_@CP (±) electrolyzer by on‐line gas chromatography (Figure S30, Supporting Information). The amounts of produced H_2_ and O_2_ collected at a current density of 100 mA cm^−2^ matches well with the theoretically calculated values, corresponding to a faradic efficiency of ≈100% (Figure 5e). The unique compatibility, high‐efficiency and good stability performance of the IrO_2_/V_2_O_5_ catalyst enable it expected to develop next‐generation water splitting technologies.

## 3 Conclusion

In summary, we have developed a simple method to synthesize highly effective IrO_2_/V_2_O_5_ catalyst for water splitting in a wide pH range. HRTEM images demonstrated clearly distorted and deformed lattice fringes of IrO_2_, corroborating the XRD and XPS results, indicating a SOSI between IrO_2_ and V_2_O_5_. The unique structure distortion endows the IrO_2_/V_2_O_5_ catalyst with a high exposure of IrO_2_ active sites. DFT and XPS indicated that the SOSI induced charge transfer from IrO_2_ to V_2_O_5_, therefore, the electrophilic IrO_2_ center can effectively promote the hydroxyl oxidation kinetics and the O—O bond coupling process, eventually promoting the fast turnover of water oxidation. Additionally, overall water‐splitting system driven by commercial AA battery (1.5 V) is also demonstrated to further promote the application. This work provides a road map in accelerating OER kinetics in different media to boost the efficiency of water electrolysis and shed light on the extension of SOSI to a variety of catalyst systems for enhancing their activity and stability.

## 4 Experimental Section

### Material

Iridium(III) chloride hydrate (IrCl_3_▪xH_2_O, 298.58), vanadium(III) chloride (VCl_3_, 157.30), and p‐phthalic acid (C_8_H_6_O_4_, 166.13) were purchased from Aladdin Inc. The commercial IrO_2_ was purchased from Sigma‐Aldrich.

### Preparation of MIL‐88B (V)

In a typical procedure, 314 mg of vanadium (III) chloride (VCl_3_) and 332 mg of terephthalic acid (C_8_H_6_O_4_) were mixed in a 50 mL beaker. Then, a solution containing 2 mL of hydrochloric acid (1 м HCl) and 10 mL of ethyl alcohol (C_2_H_5_OH) was added under vigorous stirring at room temperature. After 30 min, the resulting green suspension was further dispersed with ultrasonic treatment for 15 min. Finally, the mixture was transferred to a 50 mL Teflon‐lined stainless‐steel autoclave and heated in an electrical oven at 120 °C for 48 h. The prepared MIL‐88B (V) was placed at 50 °C under vacuum for 12 h for future use.

### Preparation of IrO_2_/V_2_O_5_ Hybrid Oxides

Typically, 50 mg IrCl_3_▪xH_2_O was dissolved in 50 mL ethanol under stirring for 30 min. After that, 0.2 g MIL‐88B (V) was slowly added into the IrCl_3_ solution under stirring. To ensure the uniform distribution of IrCl_3_, the resulting mixture was further kept stirring at room temperature for 12 h to allow the compete loading of IrCl_3_ into MIL‐88B (V) pores. After impregnation, the product was recovered by centrifugation, and washed four times with ethanol to remove IrCl_3_ remained on the outer surface of MIL‐88B (V) material. Finally, the resulting Ir(III)/MIL‐88B (V) was dried at 50 °C under vacuum for 10 h. Ir(III)/MIL‐88B (V) powder was placed in muffle furnace and heated to T (T = 300, 400, 500 °C) at a heating rate of 5 °C min^−1^ and held for 4 h. After cooling down to room temperatures, the resulting black products were collected, and denoted as IrO_2_/V_2_O_5_‐T. Notably, the typical IrO_2_/V_2_O_5_ material was obtained at 400 °C unless otherwise specified. For comparison, V_2_O_5_ was obtained by annealing MIL‐88B (V) powder at 400 °C.

### Preparation of IrO_2_/HPC and IrO_2_/V_2_O_5_‐IM

Typically, in order to ensure the consistent loading of Ir, 0.2 g hierarchically porous carbon (HPC) or synthesized V_2_O_5_ were impregnated by 6.2 mL of IrCl_3_▪xH_2_O aqueous solution (10 mg mL^−1^ based on precursor). Then water was evaporated at 60 °C and the powders were dried at 120 °C for 3 h, followed by calcination in muffle furnace at 400 °C for another 4 h.

### Characterization

Powder X‐ray diffraction patterns (PXRD) of samples were recorded using a Bruker D8 Advance diffractometer (Cu K*α*, *λ* = 1.5418 Å, operating at 40 kV/40 mA). The morphologies were characterized by transmission electron microscope (TEM, Hitachi‐7700, 100 kV) and scanning electron microscope (SEM; Hitachi S‐4800). The high‐resolution transmission electron microscope (HRTEM) were carried out by an FEI Tecnai G2 F20S‐Twin HRTEM working at 200 kV. The aberration‐corrected high‐angle annular darkfield scanning transmission electron microscope (AC HAADF‐STEM) images and the corresponding atomic‐resolution energy‐dispersive X‐ray spectroscopy element mappings were recorded by a Titan 80–300 scanning/transmission electron microscope operated at 300 kV. N_2_ adsorption–desorption measurements were carried out at 77 K using a Quantachrome SI‐MP Instrument. The surface area of the samples was estimated by method of Brunauer–Emmett–Teller (BET). Elemental analysis of Ir and V in the samples was detected by a Thermo IRIS Intrepid II inductively coupled plasma‐optical emission spectrometry (ICP‐OES). X‐ray photoemission spectroscopy were obtained on an ESCALAB MARK II spherical analyzer with a monochromatized Al K*α* source (1486.6 eV) with a base pressure in the lower 2 × 10^−7^ mbar range. The binding energy (BE) was calibrated by setting the C 1s BE to 284.8 eV with respect to the Fermi level. High‐resolution spectra were acquired with an analyzer pass energy of 40 eV. The XPS spectra were fit after subtraction of a Shirley background with the available XPSPEAK 4.1 software. In all fits, the peak separation and the peak area ratios between the Ir 4f_7/2_ and the Ir 4f_5/2_ components were constrained to 3 eV and 4:3, respectively. The detailed description about the fitting models, lineshape definition, peak position, restrained parameters summarized in the Tables S2 and S3 (Supporting Information).

### H_2_‐TPR Measurement

The mobility and redox properties of catalyst samples were evaluated via H_2_‐TPR measurement using hydrogen (H_2_) as a reductant. Briefly, 100 mg of catalyst sample with grain sizes of 60–80 mesh were loaded in a quartz reactor and pretreated with a ultrapure Ar gas (with flow rate of 30 mL min^−1^) at 200 °C for 2 h to remove the surface‐adsorbed species. The pretreated sample was then cooled to room temperature, following which the sample was heated to 800 °C under the mixed gas flow (10 vol% H_2_ in Ar with flow rate of 30 mL min^−1^) at a heating rate of 2 °C min^−1^. H_2_ consumption was measured using an online thermal conductivity detector (TCD).

### Electrochemical Measurement

All the electrochemical measurements were carried out with a three‐electrode setup using a CHI 760E electrochemical workstation (CH Instruments, Inc., Shanghai). 5 mg catalysts (IrO_2_/V_2_O_5_, V_2_O_5_, commercial IrO_2_) together with 1 mg acetylene black were dispersed in 480 µL ethanol solution with 20 µL Nafion solution (5 wt%) by sonication to form a homogeneous ink. Then, the same Ir content with 0.1 mg cm^−2^ was loaded onto the surface of a glassy carbon electrode (GCE, 5 mm diameter) to serve as the working electrode for the electrochemical tests. A saturated calomel electrode (SCE) and carbon rod were used as the reference electrode and the counter electrode, respectively. The reference electrode was also calibrated using the Pt RDE as working electrode in highly pure H_2_‐saturated electrolyte. All potentials were referenced to a reversible hydrogen electrode (RHE). For OER test, linear sweep voltammetry (LSV) was carried out in 1.0 м KOH, 1.0 м PBS, and 0.5 м H_2_SO_4_ at a scan rate of 1 mV s^−1^ after purging with O_2_ (99.999%) for at least 30 min and the HER test was also performed in accordance with above similar operations, except that O_2_ is replaced with H_2_ before the test. All the polarization curves are the steady ones after several scans with 95% iR compensation. The current density was calculated against the geometric area (0.196 cm^2^) of the electrode to obtain the specific activity. Electrochemical impedance spectroscopy (EIS) measurements were performed from 100 kHz to 0.01 Hz with an amplitude of 5 mV at an overpotential of 100 mV for HER and 300 mV for OER. The stability measurements were performed by *static* chronopotentiometry test in 1.0 м KOH, 1.0 м PBS, and 0.5 м H_2_SO_4_ and *transient* accelerated degradation test (ADT) protocol in 0.5 м H_2_SO_4_ on Au electronode (3 mm diameter, 0.0706 cm^2^_geo_). Chronopotentiometry was measured at 10 mA cm^−2^ to represent typical static long‐term stability tests. ADT tests were conducted as follows. Typically, square‐wave voltammetry consisted of 10 000 cycles were conducted between a lower potential of 0.05 V_RHE_ and an higher potential of 1.5 V_RHE_. Each cycle was maintained for 4 s. Every 2500 cycles were interrupted for LSV and CV scans (between 0.4 and 1.4 V_RHE_ at 50 mV s^−1^ to obtain the total anodic charge *Q*_anodic_ of the samples). The leaking extent Ir and V from the electrode to electrolyte was determined by ICP‐MS (NexION 2000).

The underpotential deposition of copper (Cu‐UPD) on Pt, Ru, and Ir was proven to be an ideal method for quantifying the number of active sites and the electrochemically active surface area (ECSA). Hence, the number of active sites (*n*) could require based on the generated charges during the UPD copper (Cu) stripping (Q_Cu_, Cu_upd_ → Cu^2+^ + 2e^−^), that is, *n* = Q_Cu_ / 2F.

The turnover frequency (TOF, s^−1^) was calculated by the equation: TOF = *I*/(*znF*). *F* (C mol^−1^) is the Faraday constant, *I* is the current (A) at corresponding overpotential and the factor *z* means the number of electrons required to form one hydrogen molecule or oxygen molecule (*z* = 2 for HER, *4* for OER).

The full electrolyzer configuration was assembled using two identical IrO_2_/V_2_O_5_ electrodes and measured in a two‐electrode cell with a carbon paper (CP) as the carrier. Then, the homogeneous catalyst ink was daubed uniformly in 1 cm^2^ area of CFP (precatalyst loading: 1.0 mg cm^−2^). The polarization curves were measured at a scan rate of 5 mV s^−1^ in 0.5 м H_2_SO_4_, 1.0 м KOH, and 1 м PBS solution. The stability measurements were conducted by long‐term chronoamperometry. The amount of H_2_ and O_2_ generated from water splitting was detected by online gas chromatograph (KE CHUANG GC2002) equipped with a Thermal Conductivity Detector (TCD).

### Computational Section

All DFT calculations were performed with Vienna ab‐Initio Simulation Package (VASP) in the form of Perdew–Burke–Ernzerhof (PBE) function to account for exchange‐correlation potential with the on‐site Coulomb Repulsion U term. The projector augmented wave pseudopotentials method was used for describing electron–ion interactions. In this work, the value of U was 3.0 for V and 0 for Ir. All the calculations were performed using the Cambrideg Serial Total Energy Package. The cutoff energy of 400 eV and Gaussian electron smearing method with *σ* = 0.05 eV were used. A vacuum of 15 Å was adopted along *z*‐axis. And (5 × 5 × 1) and (2 × 2 × 1) Monkhoest‐Pack *k*‐point mesh were used for all samples during electronic structure calculation and the structure optimization, respectively. During structure optimization, the geometry optimization was performed when the convergence criterion on forces became smaller than 0.02 eV Å^−1^ and the energy difference was <10^−4^ eV. The crystal orbital Hamiltonian population (COHP) calculation was performed on LOBSTER code.

The slab model of commercial IrO_2_ catalyst was constructed by cleaving the bulk structure with the stable (110) surface. The 4 × 4 supercells were employed. The atoms in the bottom two layers were kept frozen while the remaining were allowed to relax during the slab calculations. To model the IrO_2_/V_2_O_5_ catalyst, the Ir_4_O_8_ clusters supported on 4 × 4 supercell of V_2_O_5_ (001) were built. The atoms in the bottom two layers of V_2_O_5_ (001) were kept frozen while the remaining were allowed to relax during the slab calculations. The value of d‐band center was obtained by using $E_{d}=\int_{-\infty }^{\infty }N_{d}\left(\varepsilon \right)\varepsilon d\varepsilon /\int_{-\infty }^{\infty }N_{d}\left(\varepsilon \right)\varepsilon d\varepsilon$, where *ε* is the energy referring to *E*‐Fermi and *N*_d_ (*ε*) is DOS projected onto d‐states or different orbits of d‐states. The charge accumulation and depletion were obtained as *ρ*_difference_ = *ρ*_IrOx‐support_ − (*ρ*_IrOx_ + *ρ*_support_). All DFT calculations performed in this work are about the origin of the activity of three catalysts for OER. As introduced by Nørskov and coworkers,^[^ ^14^ ^]^ the acidic OER mechanism was described by a four‐electron transfer process with the following steps, where the symbol “*” represents the active site

(1) $$*+H_{2}O\to *\mathrm{OH}+H^{+}+e^{-}\Delta G_{1}$$

(2) $$*\mathrm{OH}\to *O+H^{+}+e^{-}\Delta G_{2}$$

(3) $$*O+H_{2}O\to *\mathrm{OOH}+H^{+}+e^{-}\Delta G_{3}$$

(4) $$*\mathrm{OOH}\to *+O_{2}+H^{+}+e^{-}\Delta G_{4}$$

The OER mechanism under alkaline and neutral condition can be described by the following four steps

(5) $$*+OH^{-}\to *\mathrm{OH}+e^{-}\Delta G_{1}$$

(6) $$*\mathrm{OH}+OH^{-}\to *O+H_{2}O+e^{-}\Delta G_{2}$$

(7) $$*O+OH^{-}\to *\mathrm{OOH}+e^{-}\Delta G_{3}$$

(8) $$\mathrm{OOH}*+OH^{-}\to *+O_{2}+H_{2}O+e^{-}\Delta G_{4}$$

The adsorption energy (*E*_ads_) of species X is calculated by *E*_ads_ = *E*(X/slab) – *E*(X) – *E*(slab). The thermodynamic free energies were obtained as *G* = *E*_DFT_ + *E*_ZPE_ – *TS*, where *E*_DFT_ is the DFT ground state, *E*_ZPE_ is zero‐point energy, and *S* is the entropy. Free energies were used for all absorbed species (*OH, *O, and *OOH), and free energy correction values for adsorbates are provided in Table S10 (Supporting Information). The free energy of gaseous O_2_ is derived as *G*(O_2_) = 2*G*(H_2_O) − 2*G*(H_2_) + 4.92 (eV). The free energy at an applied overpotential U is calculated as *G*_U_ = *G* − *neU*, where *n* is the number of (H^+^ + e^−^) pairs involved and *e* is the transferred electron. More precisely, the catalytic performance was estimated by the magnitude of the potential‐determining step for the OER, *G* ^OER^. This was the specific reaction step in the four‐step mechanism with the largest Δ*G*

(9) $$G^{\mathrm{OER}}=\max\left[\Delta G_{1},\Delta G_{2},\Delta G_{3},\Delta G_{3}\right]$$

The theoretical overpotential, which is independent of pH, at standard conditions is then given by following equation

(10) $$\eta^{\mathrm{OER}}=\left(G^{\mathrm{OER}}/e\right)-1.23V$$

## Conflict of Interest

The authors declare no conflict of interest.

## Supporting information

Supporting Information

Supplemental Movie 1

## Data Availability

Research data are not shared.
