# Supplementary material for: Strong Oxide‐Support Interaction over IrO2/V2O5 for Efficient pH‐Universal Water Splitting
Source: Adv Sci (Weinh). 2022 Feb 12;9(11):2104636. doi: 10.1002/advs.202104636 (PMC9008424; doi:10.1002/advs.202104636)
Supplement: Supplementary file 1 — Supporting Information [file ADVS-9-2104636-s001.pdf]

ADVANCED SCIENCE
Open Access

Supporting Information

for *Adv. Sci.*, DOI 10.1002/advs.202104636

Strong Oxide-Support Interaction over IrO_2/V_2O_5 for Efficient pH-Universal Water Splitting

*Xiaozhong Zheng, Minkai Qin, Shuangxiu Ma, Yuzhuo Chen, Honghui Ning, Rui Yang, Shanjun Mao and Yong Wang**

WILEY-VCH

ADVANCED SCIENCE
Open Access

Supporting Information

for *Adv. Sci.*, DOI: 10.1002/advs.202104636

Strong Oxide-Support Interaction over IrO_2/V_2O_5 for Efficient pH-Universal Water Splitting

*Xiaozhong Zheng, Minkai Qin, Shuangxiu Ma, Yuzhuo Chen, Honghui Ning, Rui Yang, Shanjun Mao, and Yong Wang**

Supporting Information

Strong Oxide-Support Interaction over IrO_2/V_2O_5 for Efficient pH-Universal Water Splitting

Xiaozhong Zheng, Minkai Qin, Shuangxiu Ma, Yuzhuo Chen, Honghui Ning, Rui Yang, Shanjun Mao, and Yong Wang[*]

X. Zheng, M. Qin, S. Ma, Y. Chen, H. Ning, Dr. R. Yang, Dr. S. Mao, Prof. Y. Wang

Advanced Materials and Catalysis Group, State Key Laboratory of Clean Energy Utilization, Center of Chemistry for Frontier Technologies, Institute of Catalysis, Department of Chemistry, Zhejiang University, Hangzhou, 310028, P. R. China.

Prof. Dr. Y. Wang

College of Chemistry and Molecular Engineering, Zhengzhou University, Zhengzhou 450001, China.

E-mail: chemwy@zju.edu.cn

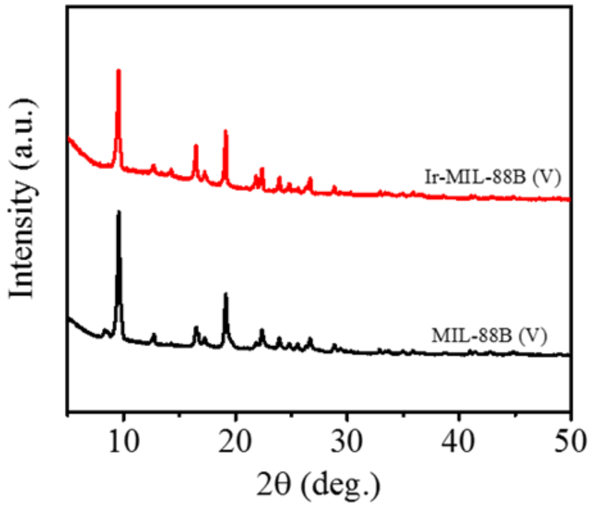

Figure S1. XRD patterns of MIL-88B (V) and Ir-MIL-88B (V).

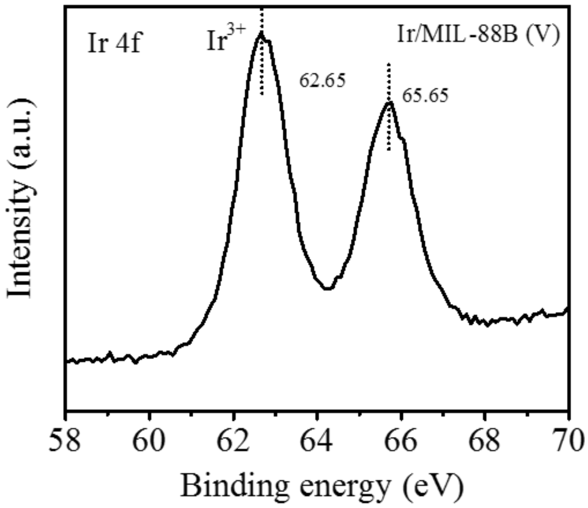

Figure R2. Ir 4f spectra of and Ir/MIL-88B (V).

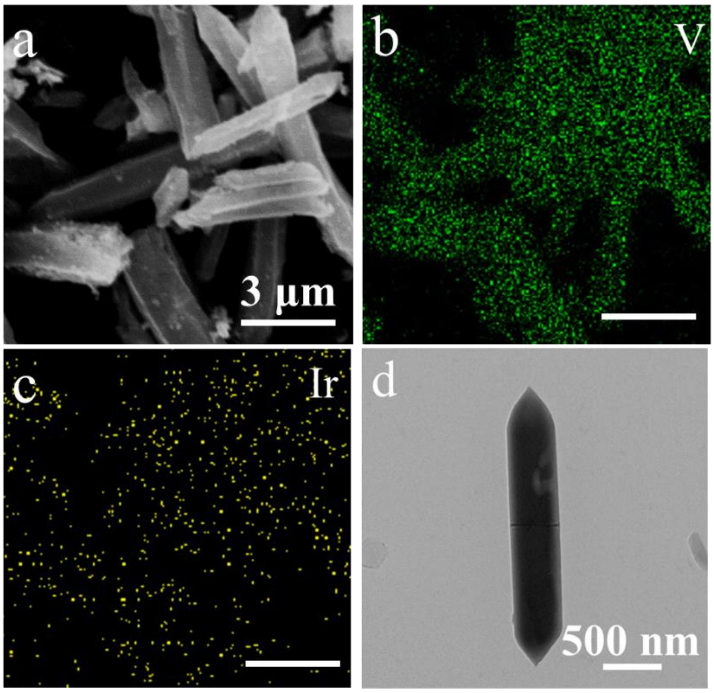

Figure S3. (a-c) SEM image of Ir-MIL-88B (V) and corresponding elemental mappings (V and Ir element). (d) TEM image of Ir-MIL-88B (V).

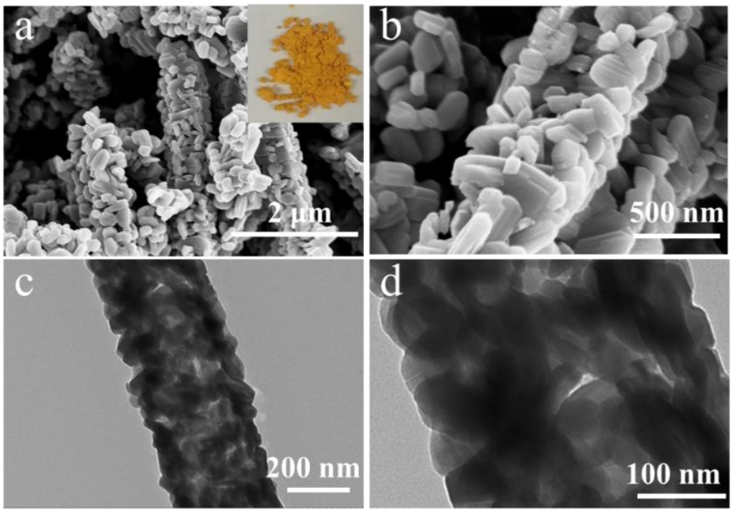

Figure S4. SEM (a, b) and TEM (c, d) images of V_2O_5; the insert in a is the digital photo of V_2O_5.

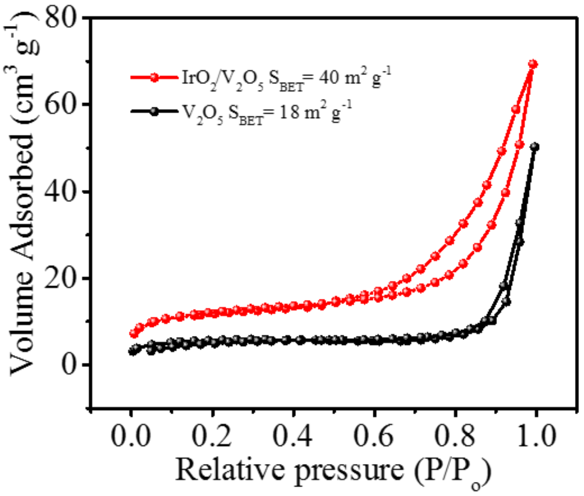

Figure S5. Nitrogen adsorption-desorption isotherms of IrO_2/V_2O_5 and V_2O_5, respectively.

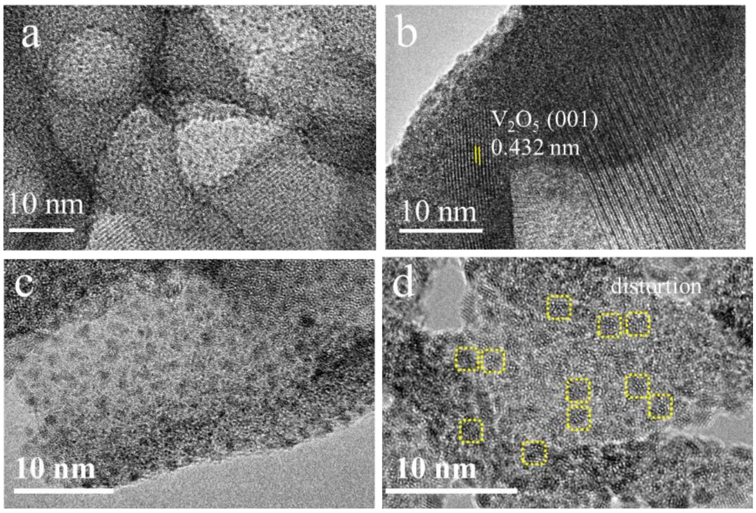

Figure S6. HRTEM images (a-d) of IrO_2/V_2O_5-400 for measuring the size distribution.

Clear lattice fringes with interplanar distances of 0.432 nm was observed, corresponding to the (001) and planes of crystalline V_2O_5, and can clearly find distroted and deformed iridium oxides nanoclusters embedded on it.

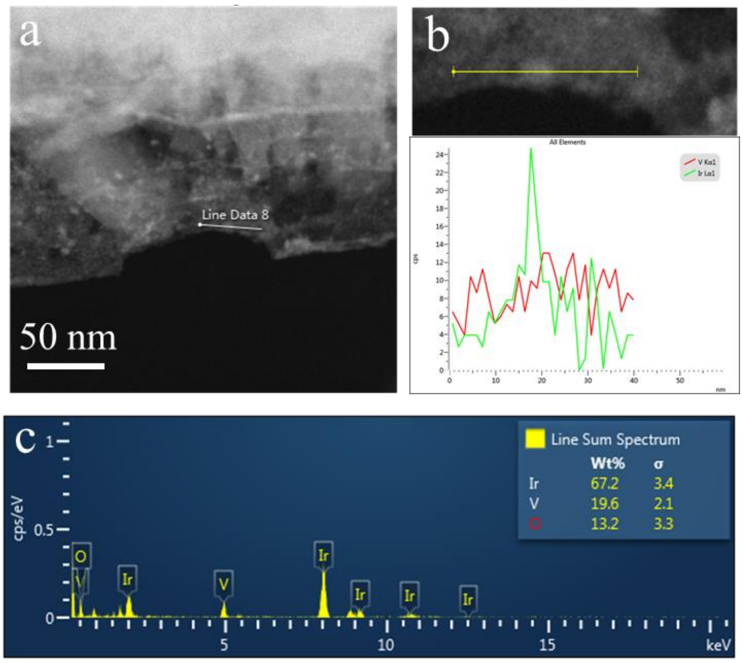

Figure S7 (a) The STEM image and (b, c) composition and elemental distribution of IrO_2/V_2O_5 catalyst by line-scanning.

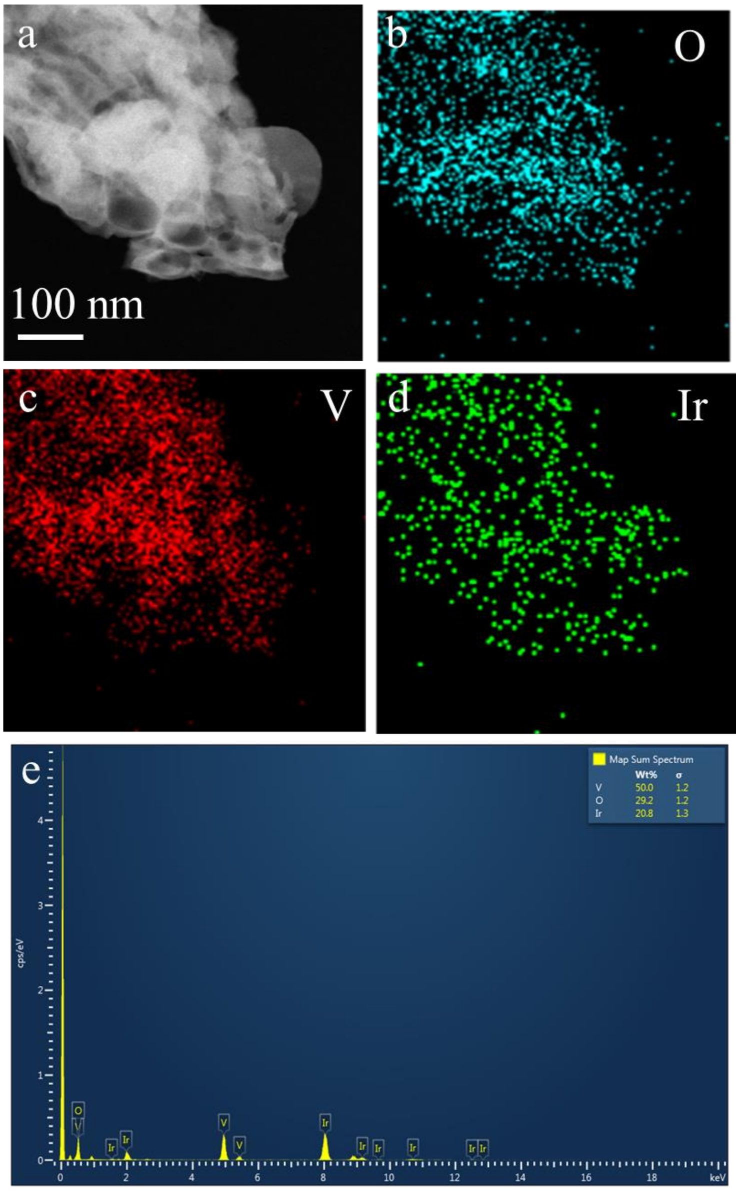

Figure S8. (a-d) HAADF-STEM image and corresponding EDS mappings of IrO_2/V_2O_5 for Ir, V and O; (e) corresponding element content.

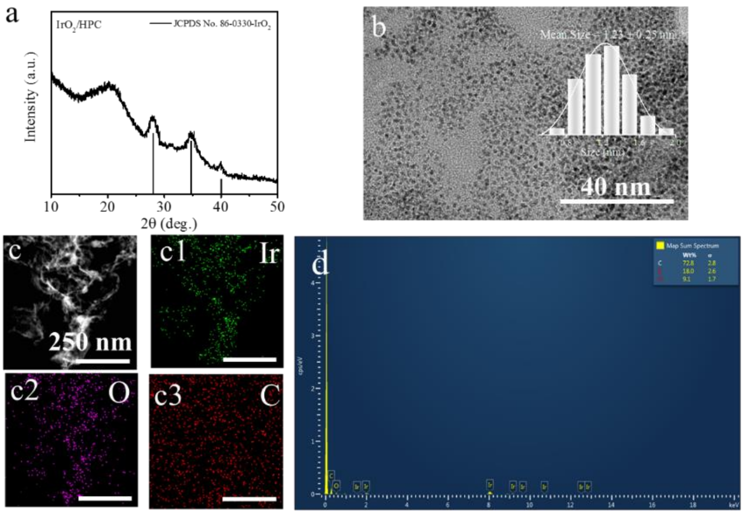

Figure S9. (a) XRD pattern of IrO_2/HPC. (b) The TEM image of IrO_2/HPC, the insert in b image is the size distributing patter. (c-c3) HAADF-STEM image and EDS mappings of IrO_2/HPC for Ir, O and C. (d) corresponding element content.

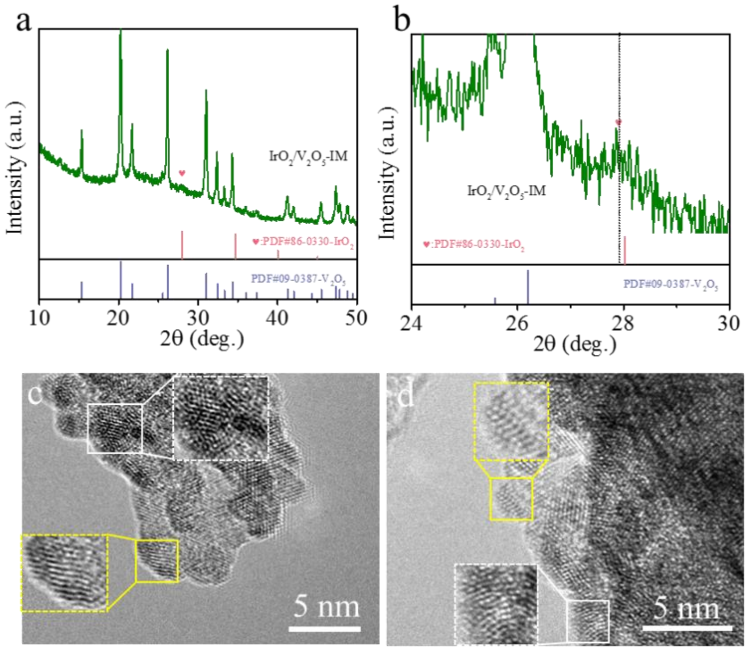

Figure S10. (a, b) XRD pattern and (c, d) HRTEM images of IrO_2/V_2O_5-IM.

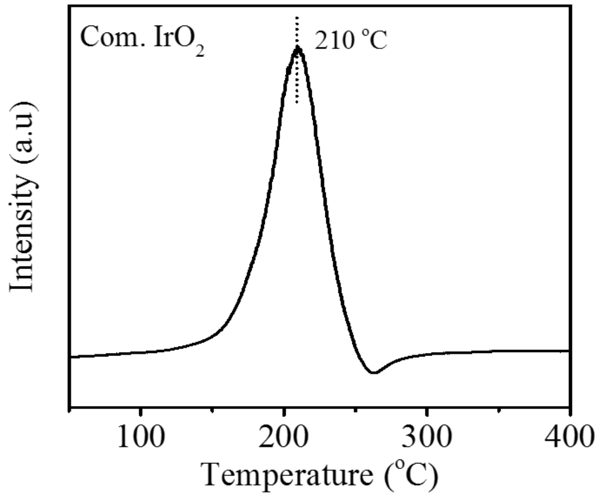

Figure S11. H_2-TPR pattern of commercial IrO_2.

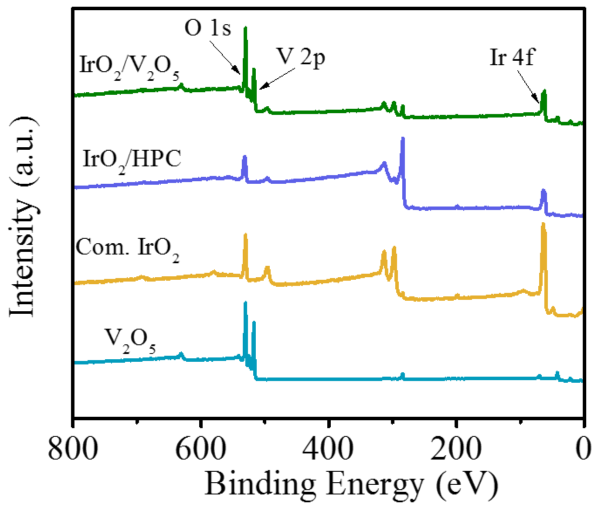

Figure S12. XPS survey of IrO_2/V_2O_5, IrO_2/HPC, commercial IrO_2 and V_2O_5.

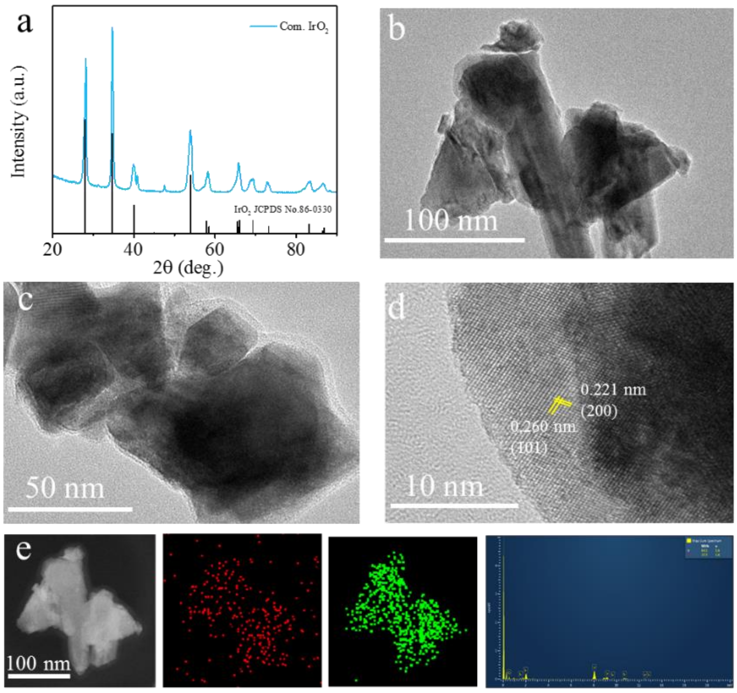

Figure S 13. (a) The XRD pattern and (b-d) TEM and HRTEM images of commercial IrO_2 sample; (e) HAADF-STEM image and corresponding EDS mappings of commercial IrO_2 for Ir and O, and corresponding element content.

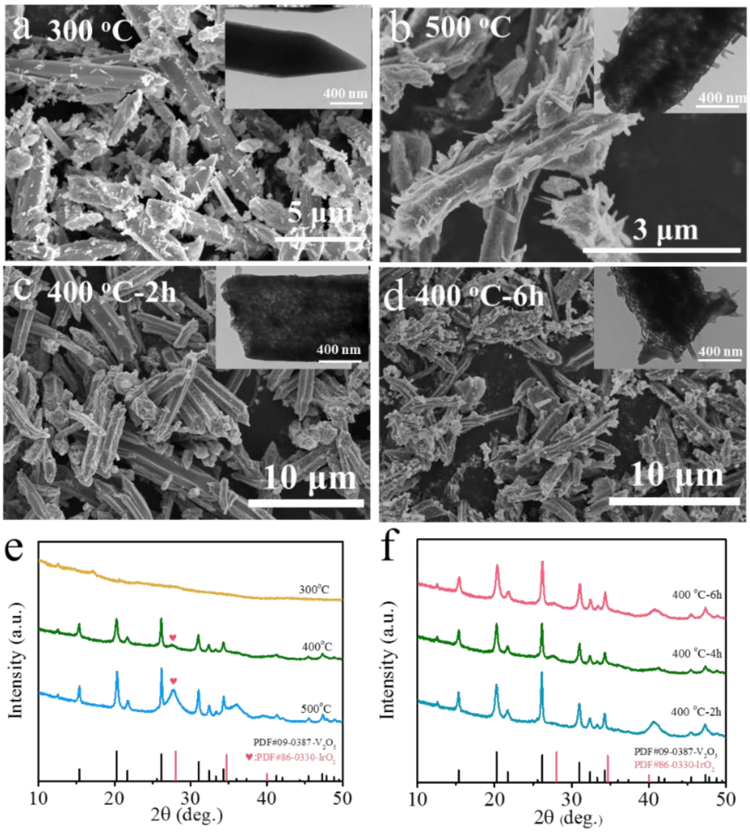

Figure S14. (a-b) SEM and TEM images of IrO_2/V_2O_5-T (T=300,500 ^oC). (c-d) SEM and TEM images of IrO_2/V_2O_5-400 with different calcination time (2 and 6 h). (e) XRD patterns of IrO_2/V_2O_5-T (T=300, 400 and 500 ^oC) and (f) different calcination time (2, 4 and 6 h) of IrO_2/V_2O_5-400.

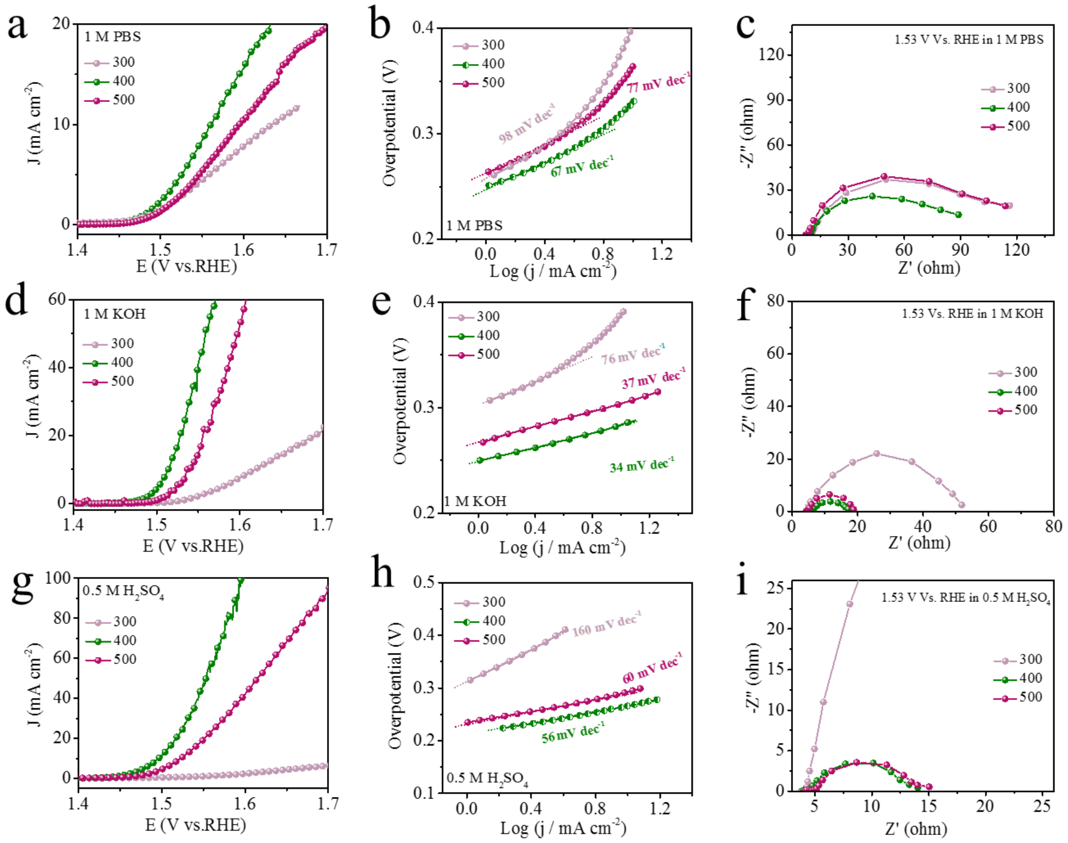

Figure S15. OER electrochemical test of IrO_2/V_2O_5-T (T = 300, 400 and 500 ^{o}C). IrO_2/V_2O_5-400 electrocatalyst shows best OER performance in all-pH range, therefore, we selected the IrO_2/V_2O_5-400 as the optimized catalyst for further investigation, which was simply denoted as IrO_2/V_2O_5 in the manuscript.

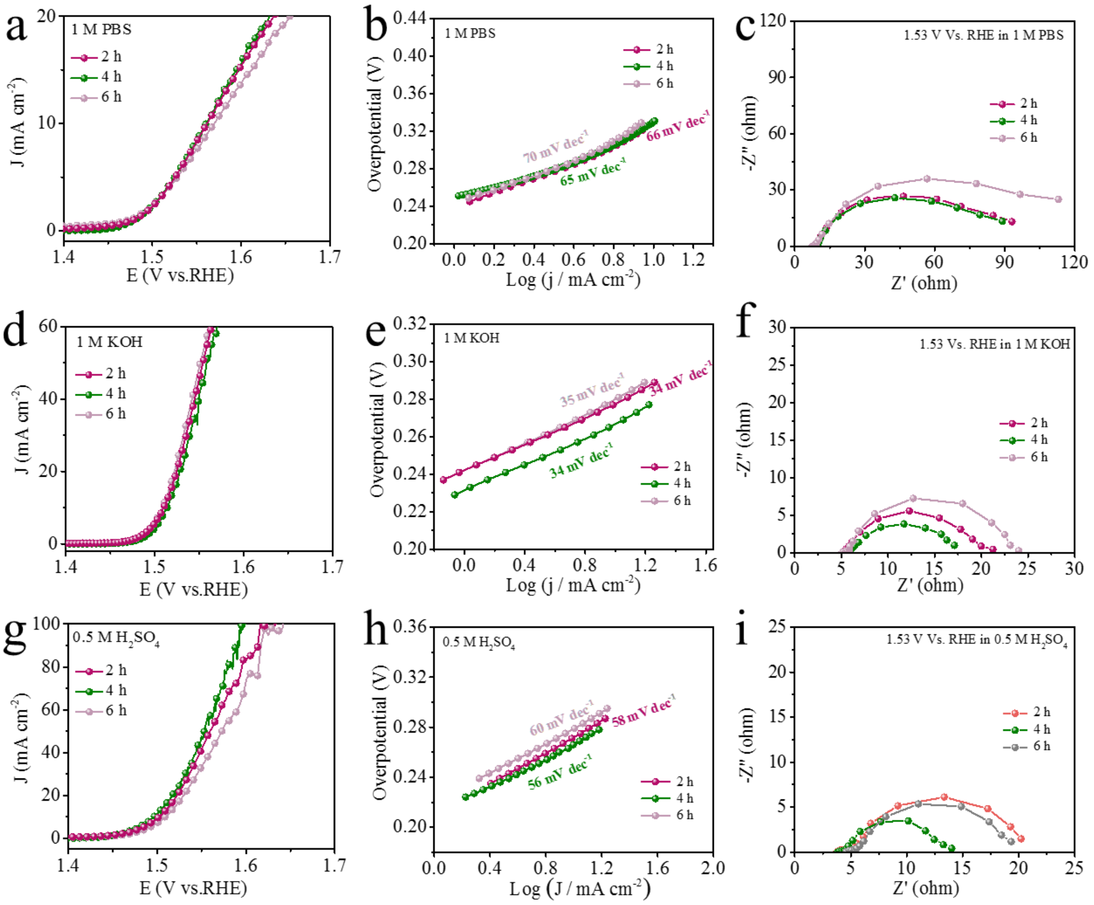

Figure S16. OER electrochemical test of IrO_2/V_2O_5-400 for different calcination time (2, 4 and 6 h).

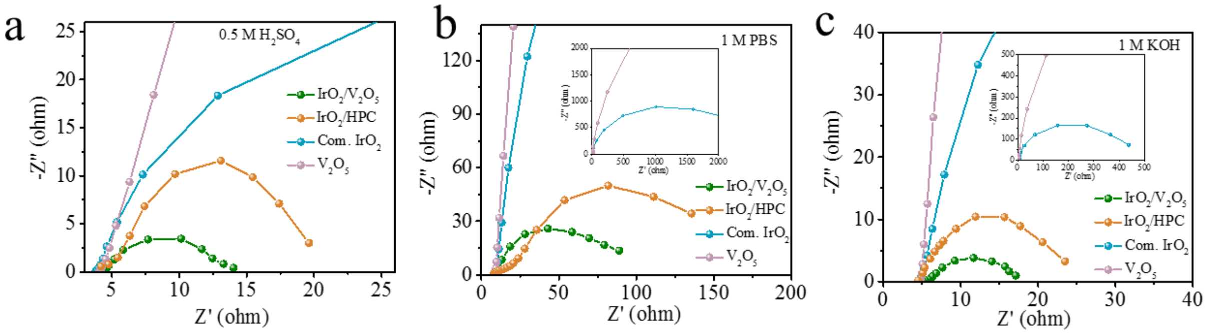

Figure S17. Electrochemical impedance spectroscopy (EIS) analyses of of IrO_2/V_2O_5, IrO_2/HPC, commercial IrO_2 and V_2O_5 at 1.53 V Vs. RHE with 5 mV AC potential from 10 kHz to 0.01 Hz for OER in 0.5 M H_2SO_4 (a), 1 M PBS (b) and 1 M KOH (c), respectively.

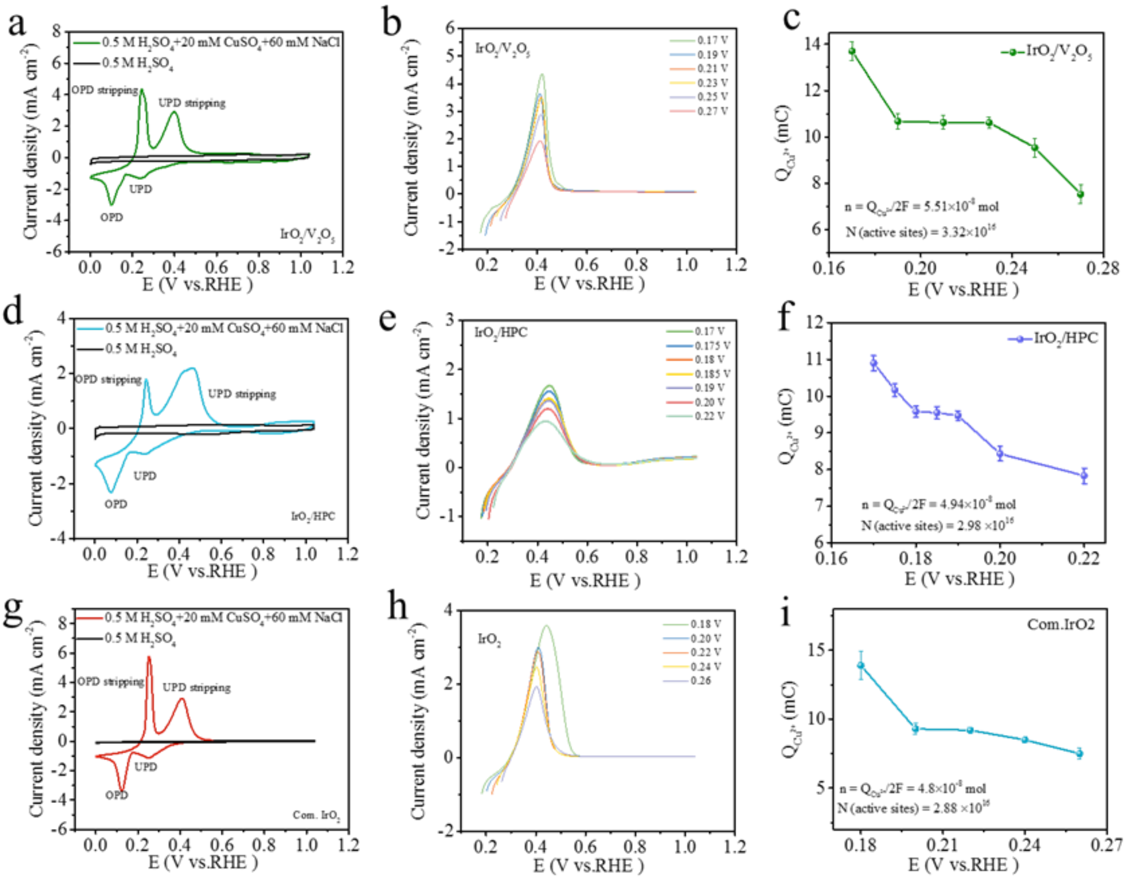

Figure S18. The cyclic voltammetry scans in different solutions, LSV curves under different initial voltages and the corresponding charges required to strip the Cu deposited at different under potentials for the IrO_2/V_2O_5 (a-c), IrO_2/HPC (d-f) and commercial IrO_2 (h-j) with same Ir loading 0.1 mg/cm^2.

Supplement: The number of active sites in electrocatalysts was evaluated by the underpotential deposition (UPD) of copper on Ir, Ru and Pt materials. The Cu UPD method is based on the principle that the active sites for the reduction of protons are also effective for the reduction of Cu^{2+} ions at an underpotential condition. Therefore, the charge required for oxidative stripping of copper produced during UPD process can be employed to estimate the number of active sites. Cl^- ion is an ideal additive to resolve the UPD and OPD peaks because it can absorb on the UPD allayer instantly and thus inhibit the OPD process. Therefore, in a 0.5 M H_2SO_4 + 20 mM $CuSO_4$ + 60 mM NaCl solution, peaks of UPD, OPD and the corresponding stripping are distinct in CV curves, followed by the deposition of copper at various underpotentials (from 0.17 V to 0.27 V) for 120 s in the same solution. A linear voltammetry scan (2 mV/s) was then performed from the set underpotential to 1.04 V_{RHE} at which all of the UPD copper had been removed, and the $Q_{stripping}$ was recorded. Ideally the set underpotential needs to reach the edge of over potential deposition region of Cu and the higher potential region of the UPD of Cu.

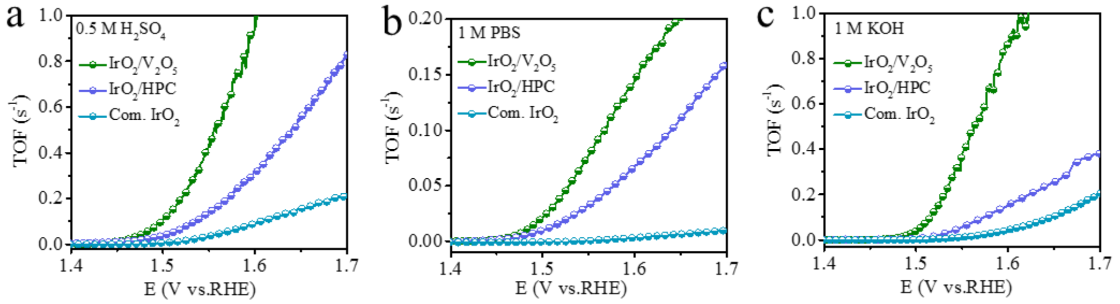

Figure S19. TOF values (a-c) of IrO_2/V_2O_5, IrO_2/HPC and IrO_2 and for OER in 0.5 M H_2SO_4, 1 M PBS and 1 M KOH, respectively.

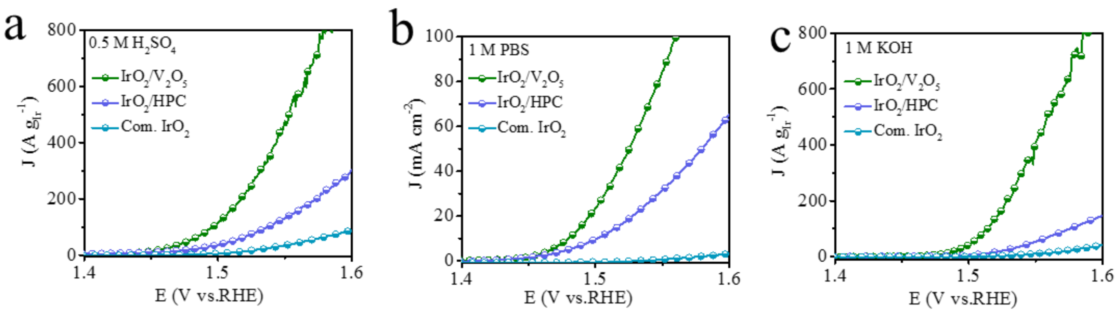

Figure S20. Mass activity based on ICP-OES (a-c) of IrO_2/V_2O_5, IrO_2/HPC and IrO_2 and for OER in 0.5 M H_2SO_4, 1 M PBS and 1 M KOH, respectively.

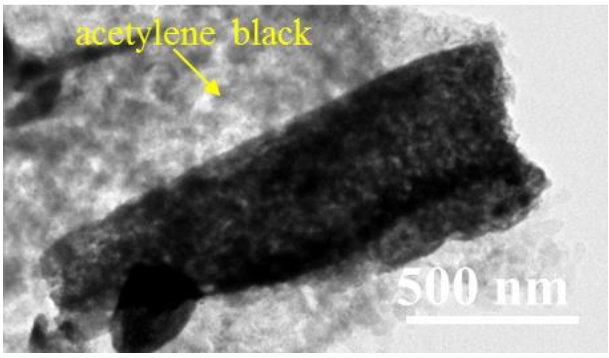

Figure S21. The TEM image of IrO_2/V_2O_5 after stability.

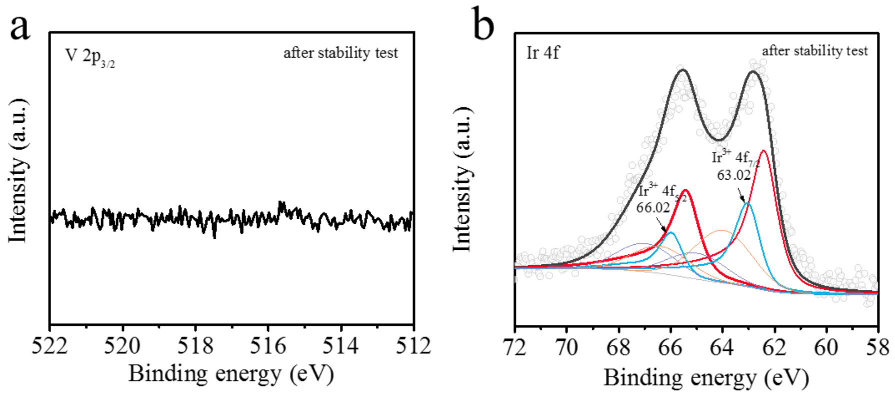

Figure S22. The V2$p_{3/2}$ (a) and Ir4f (b) spectra of IrO_2/V_2O_5 after stability test.

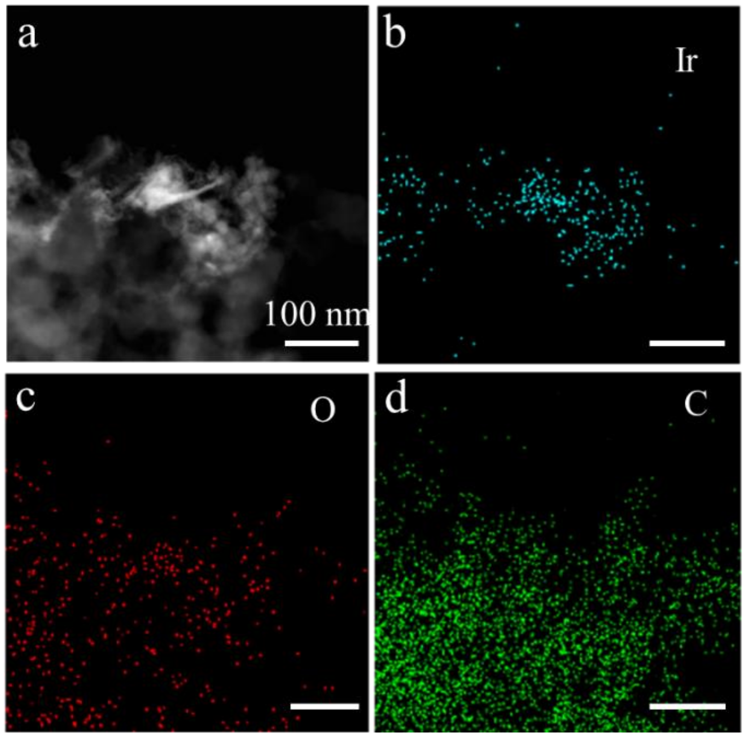

Figure S23. (a-d) HAADF-STEM image and corresponding EDS mappings of IrO_2/HPC for Ir, O and C after stability test.

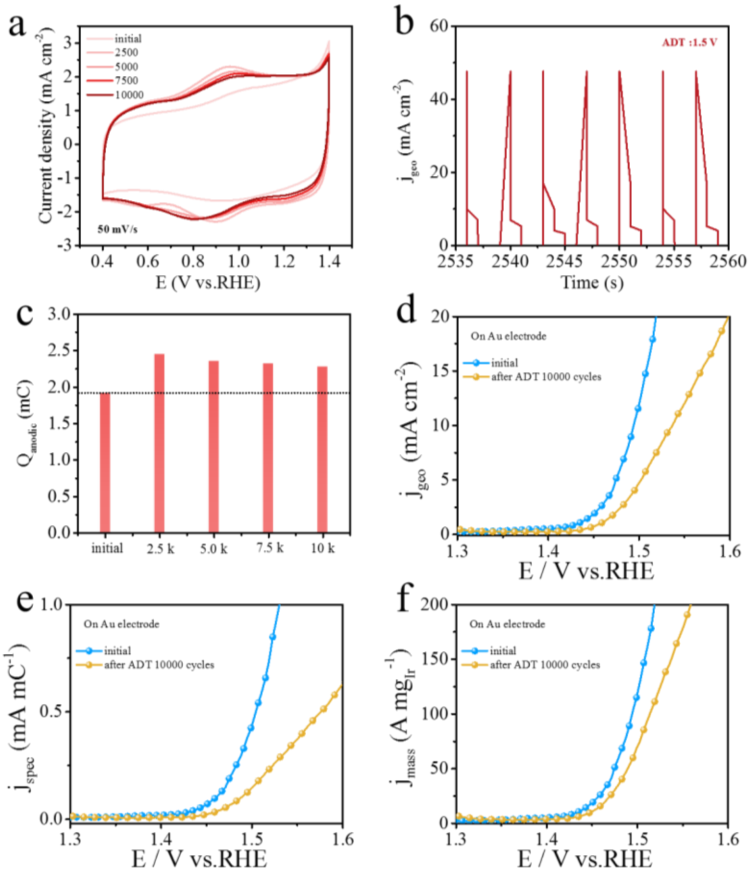

Figure S24. Electrochemical characteristics of IrO_2/V_2O_5-ADT-1.5V. (a) the change of surface redox activity during CV cycling; (b) the current densities (geometric) during the ADT, c) Evolution of Q anodic obtained from CVs as well as OER performance degradation including (d) j_{geo}, (e)j_{spec} and (f)j_{mass}.

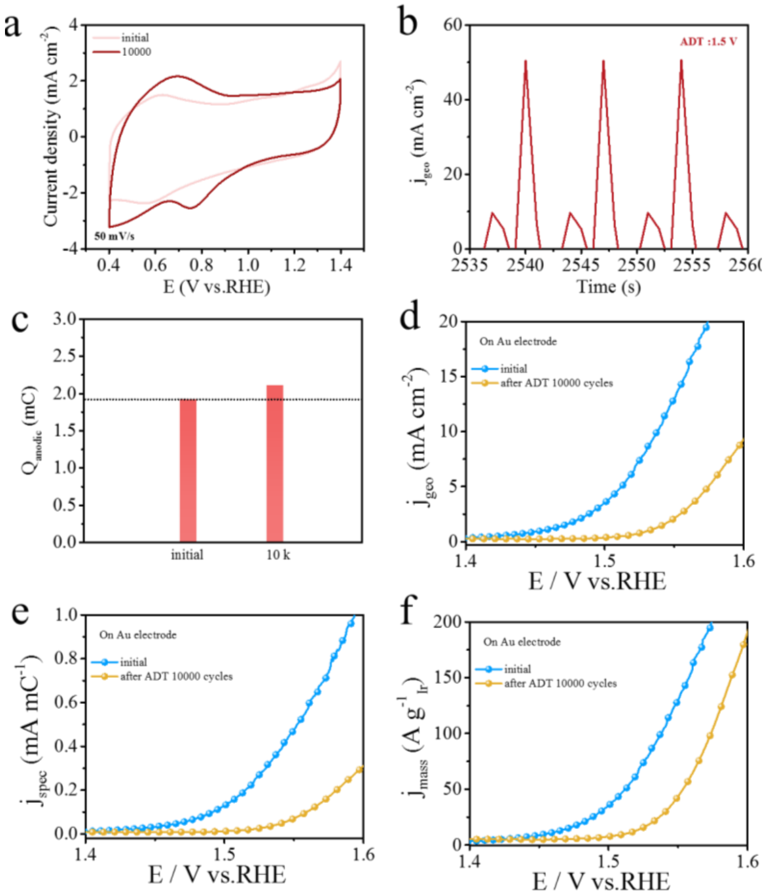

Figure S25. Electrochemical characteristics of IrO_2/HPC-ADT-1.5V. (a) the change of surface redox activity during CV cycling; (b) the current densities (geometric) during the ADT, c) Evolution of Q anodic obtained from CVs as well as OER performance degradation including (d) j_{geo}, (e)j_{spec} and (f)j_{mass}.

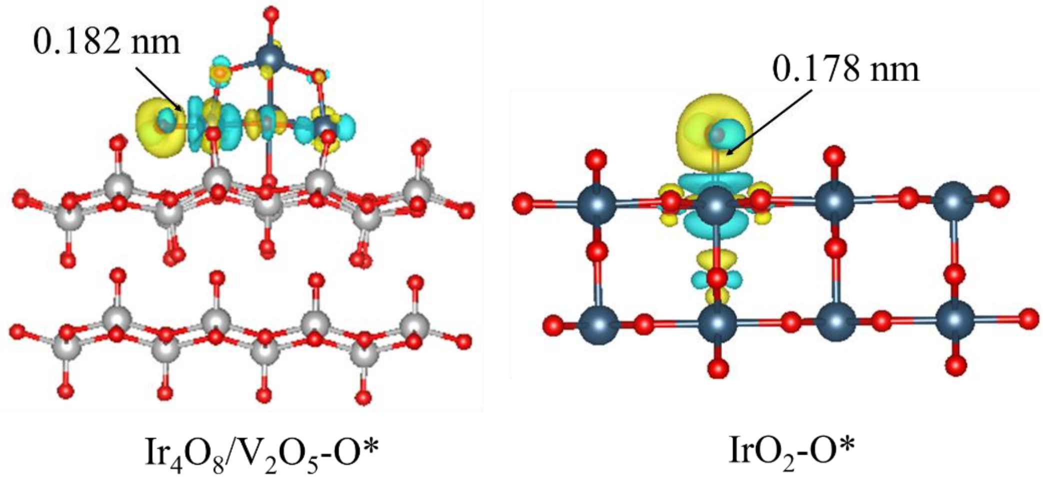

Figure S26. The bond length of Ir-O* on (a) Ir_4O_8/V_2O_5 and (b) IrO_2.

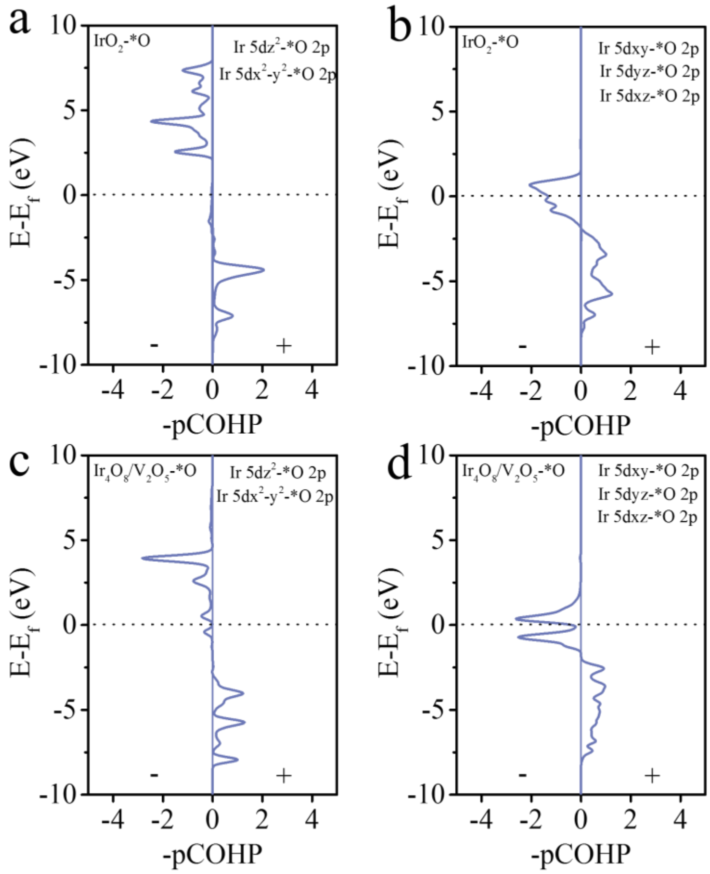

Figure S27. –pCOHP curves of Ir 5d (e_g, t_{2g}) orbitals interaction with O 2p orbital in Ir_4O_8/V_2O_5 (a, b) and rutile IrO_2 (c, d).

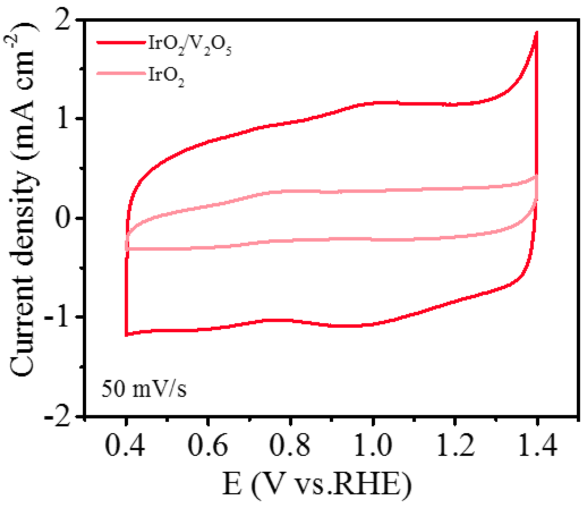

Figure S28. Cyclic voltammetry (CV) curves of IrO_2/V_2O_5 and commercial IrO_2 in 0.5 M H_2SO_4 electrolyte.

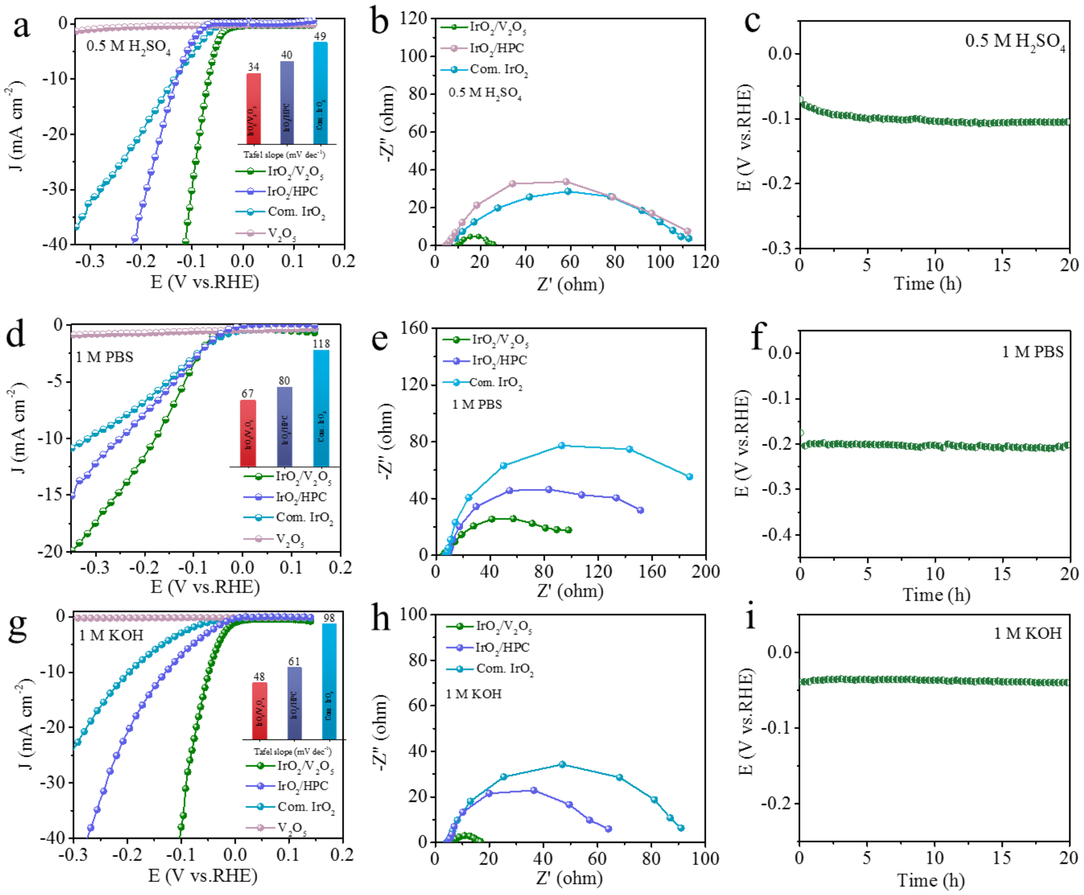

Figure S29. HER electrochemical test of IrO_2/V_2O_5, IrO_2/HPC, commercial IrO_2 and V_2O_5 in 0.5 M H_2SO_4, 1 M PBS and 1 M KOH, respectively. (a) The polarization curves, (b) EIS analyses at -0.1 V Vs. RHE and (c) chronopotentiometry tests at 10 mA cm^{-2} current density in 0.5 M H_2SO_4. (d) The polarization curves, (e) EIS analyses at -0.1 V Vs. RHE and (f) chronopotentiometry tests at 10 mA cm^{-2} current density in 1M PBS. (g) The polarization curves, (h) EIS analyses at -0.1 V Vs. RHE and (i) chronopotentiometry tests at 10 mA cm^{-2} current density in 1 M KOH.

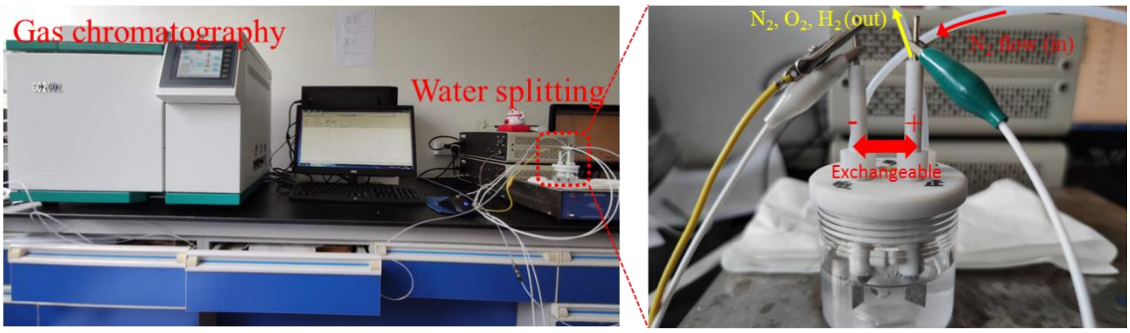

Figure S30. The digital picture of water splitting coupled with on-line gas chromatograph (GC) for measuring the amount of H_2 and O_2.

Table S1. ICP-OES analysis for the prepared IrO_2/V_2O_5, IrO_2/HPC.

	Ir (wt%)	V (wt%)
IrO_2/V_2O_5	19.7	41.4
IrO_2/HPC	19.1	-
Commercial IrO_2	Theoretical content: 85.0	-

Supplement:

Sample preparation: 5.3 mg IrO_2/V_2O_5 or IrO_2/HPC samples were treated at 350 ^oC for 6 h under H_2 atmosphere to fully reduce iridium oxides to metal iridium. The resultant powder was added to the aqua regia (a mixture of 9 ml HCl and 3 ml HNO_3). Finally, the mixture was transferred to a 50 mL Teflon-lined stainless-steel autoclave and heated in an electrical oven at 180 °C for 24 h. Then the obtained solution is diluted to 250 ml, and the Ir loading of sample is determined by ICP-OES.

Table S2: Fit parameters for Ir 4f of commercial IrO_2, IrO_2/HPC and IrO_2/V_2O_5.

Com. IrO_2	Ir $4f_{7/2}$	Ir $4f_{5/2}$	Ir $4f_{7/2}$ sta 1	Ir $4f_{5/2}$ sta 1	Ir $4f_{5/2}$ sta 2
line shape	DS(0.2,230) SGL(55)	DS(0.2,230) SGL(55)	GL(0)	GL(0)	GL(0)
FWHM (eV)	0.9	0.9	2.6	2.6	2.6
B.E. (eV)	61.95	64.95	63.05	66.05	68.05
IrO_2/HPC	Ir $4f_{7/2}$	Ir $4f_{5/2}$	Ir $4f_{7/2}$ sta 1	Ir $4f_{5/2}$ sta 1	Ir $4f_{5/2}$ sta 2
line shape	DS(0.2,230) SGL(55)	DS(0.2,230) SGL(55)	GL(0)	GL(0)	GL(0)
FWHM (eV)	1.0	1.0	2.6	2.6	2.6
B.E. (eV)	61.98	64.98	62.89	65.82	68.1
IrO_2/V_2O_5	Ir $4f_{7/2}$	Ir $4f_{5/2}$	Ir $4f_{7/2}$ sta 1	Ir $4f_{5/2}$ sta 1	
line shape	DS(0.1,230) SGL(55)	DS(0.1,230) SGL(55)	GL(0)	GL(0)	
FWHM (eV)	0.96	0.96	2.6	2.6	

B.E. (eV)	62.41	65.41	63.80	66.23

Note: DS(α,n) is a Doniach-Šunjic profile with an asymmetry parameter that is convoluted with a Gaussian with a width; SGL(m) whose ratio is given by the parameter m (0 pure Gaussian, 100 pure Lorentzian).

Table S3: Fit parameters for V $2p_{3/2}$ of IrO_2/V_2O_5 and V_2O_5.

IrO_2/V_2O_5	V $2p_{3/2}$ (V)	V $2p_{3/2}$ (IV)
line shape	GL(60)	GL(60)
FWHM (eV)	1.48	1.18
B.E. (eV)	517.30	516.05
V_2O_5	V $2p_{3/2}$ (V)	V $2p_{3/2}$ (IV)
line shape	GL(60)	GL(60)
FWHM (eV)	1.28	1.0
B.E. (eV)	517.36	516.11

Table S4. Comparison of OER activities with various recently reported state-of-the-art catalysts in acidic electrolyte.

**Electrocatalysts**	$\eta_{10\ mA\ cm^{-2}}$ **/ mV**	**Tafel slope / mV dec^{-1}**	**Electrolyte**	**References**
**IrO_2/V_2O_5**	266	56	0.5 M H_2SO_4	This work
**La_3IrO_7**	296	52	0.1 M $HClO_4$	*Adv. Energy Mater.* **2020**, 2003561
**$IrO_x/SrIrO_3$**	270-290	40	0.5 M H_2SO_4	*Science.* **2016**, 353, 1011
**DO-$IrTe_2$**	298	49.2	0.5 M H_2SO_4	*Adv. Funct. Mater.* **2020**, 30, 2004375
**Ru-N-C**	267	52.6	0.5 M H_2SO_4	*Nat. Commun.* **2019**, 10, 4849
**$W_{1-x}Ir_xO_{3-\delta}$**	370	125	1 M H_2SO_4	*Energy Environ. Sci.* **2017**, 10, 2432
**Ru@IrO_x**	282	69	0.05 M H_2SO_4	*Chem* **2019**,5, 445–459
**Co-RuIr**	235	66.9	0.1 M $HClO_4$	*Adv. Mater.* **2019**, 31, 1900510
**Ir-$NiCo_2O_4$**	240	60	0.5 M H_2SO_4	*J. Am. Chem. Soc.* **2020**, 142, 18378
**Ir-Ag NTS**	285	61.1	0.5 M H_2SO_4	*Nano Energy* **2019**, 56, 330
**Ir/GF**	290	46	0.5 M H_2SO_4	*Nano Energy* **2017**, 40, 27
**RuO_2 NS**	255	38	0.1 M $HClO_4$	*Adv. Energy Mater.* **2019**, 9, 1803795
**Ir WNWs**	270	43.6	0.1 M $HClO_4$	*Nanoscale*, **2018**, 10, 1892
**Ba_2YIrO_6**	330	67	0.1 M $HClO_4$	*Nat. Commun.* **2016**, 7, 12363
**FeN_4/NF/EG**	294	129	0.5 M H_2SO_4	*Adv. Energy Mater.* **2018**, 8, 1801912

**IrW/C**	310	56.6	0.1 M $HClO_4$	*ACS Cent. Sci.* **2018**, 4, 1244

Table S5. Comparison of OER activities with various recently reported state-of-the-art catalysts in neutral electrolyte.

**Electrocatalysts**	$\eta_{10\ mA\ cm^{-2}}$ **/ mV**	**Tafel slope / mV dec^{-1}**	**Electrolyte**	**References**
**IrO_2/V_2O_5**	329	67	1 M PBS	This work
**CoIr-0.2**	373	117.5	1 M PBS	*Adv. Mater.* **2018**, 30, 1707522
**$IrSe_2$**	315	-	1 M PBS	*Angew. Chem. Int. Ed.* ***2019****, 58, 14764*
**IrO_x/CN_xNTS**	472	170	0.1 M $KHCO_3$	*J. Mater. Sci.* **2018**, 53**,** 4939
**$Co_3(PO_4)_2$**	370	70	0.1 M PBS	*Angew. Chem. Int. Ed.* **2019**, 58, 14599
**Co-Bi/Ti**	469	108	0.1 M KBi	*J. Mater. Chem. A* **2017**, 5, 7305
**Ir-NSG**	307	74.2	1 M PBS	*Nat. Commun.* **2020**, 11, 4246
**$Ni_{0.1}Fe_{0.9}P$**	560	133	1 M PBS	*Angew. Chem. Int. Ed.* **2018**, 130, 15671
**CoO/Co_4N**	398	83	1 M PBS	*J. Mater. Chem. A* **2018**, 6, 24767
**Co-Pi NA/Ti**	450	187	0.1 M PBS	*Angew. Chem. Int. Ed.* **2017**, 56, 1064
**$(Fe_{0.5}Ni_{0.5})_2P$/NF**	396	182	0.1 M PBS	*Nano Energy* **2017**, 38, 553
**Co_3O_4 QDs**	490	80	0.2 M PBS	*Ind. Eng. Chem. Res.* **2018**, 57, 1441
**Co4Mo**	490	144	0.1 M PBS	*Angew. Chem. Int. Ed.* **2019**, 131, 145
**NiCoFeP**	330	60	0.5 M $KHCO_3$	, *Nat. Chem.* **2017**, 10, 149
**CoMoNiS-NF-31**	405	71	1 M PBS	*J. Am. Chem. Soc.* **2019**, 141, 10417
**Ni-Fe-Mg**	360	150	0.5 M $KHCO_3$	*Adv. Mater.* **2020**, 32, 1906806

Table S6. Comparison of OER activities with various recently reported state-of-the-art catalysts in alkaline electrolyte.

**Electrocatalysts**	$\eta_{10\ mA\ cm^{-2}}$ **/ mV**	**Tafel slope / mV dec^{-1}**	**Electrolyte**	**References**
**IrO_2/V_2O_5**	283	34	1 M KOH	This work
**N-CoS**	240	98	1 M KOH	*ACS Catal.* **2017**, 7, 4214
**$CoSe_{1.26}P_{1.42}$**	255	87	1 M KOH	*ACS Energy Lett.* **2019**, 4, 987
**Ni_3S_2/MnO_2/NF**	260	61	1 M KOH	, *Appl. Catal. B-Environ.* **2019**, 254 ,329
**Co_3O_4/CeO_4**	270	60	1 M KOH	*Adv. Mater.* **2019**, 31, 1900062

**N-NiMoO$_4$/NiS$_2$**	283	44.3	1 M KOH	*Adv. Funct. Mater.* **2019**, 29, 1805298
**LaFe$_{0.2}$Ni$_{0.8}$O$_3$**	302	50	1 M KOH	*Angew. Chem. Int. Ed.* **2019**, 58, 2316
**NiO/Co$_3$O$_4$**	262	58	1 M KOH	*ACS Catal.* **2020**, 10, 12376
**Ni–Fe–MOF NSs**	221	56	1 M KOH	*Angew.Chem. Int.Ed.* **2019**, 58, 7051
**Ir 18wt% -NiO**	215	35	1 M KOH	*J. Am. Chem. Soc.* **2020**, 142, 7425
**Fe$_x$Ni$_{1-x}$O**	297	37	0.5 M KOH	*ACS nano* **2015**, 9, 5180
**NiFe LDH**	230	47	1 M KOH	*Adv. Energy Mater.* **2019**, 9, 1900881
**a-Co$_4$Fe(OH)$_x$**	295	52	1 M KOH	*J. Mater. Chem. A*, **2017**, 5, 1078

Table S7. The OER stability of Ir-based electrocatalyst reported in literatures.

Electrocatalysts	Stability	Electrolyte	References
**IrO$_2$/V$_2$O$_5$**	20 hours at 10 mA/cm$^2_{geo}$	0.5 M H$_2$SO$_4$<br>1 M KOH<br>1 M PBS	This work
**La$_3$IrO$_7$**	60000 s at 10 mA/cm$^2_{geo}$	0.1 M HClO$_4$	*Adv. Energy Mater.* **2020**, 2003561
**IrO$_x$/SrIrO$_3$**	30 hours at 10 mA/cm$^2_{geo}$	0.5 M H$_2$SO$_4$	*Science.* **2016**, 353, 1011
**Ir-NSG**	6000s at 45.47 mA mg Ir^{-1}	0.1 M HClO$_4$	*Nat. Commun.* **2020**, 11, 4246
**W$_{1-x}$Ir$_x$O$_{3-δ}$**	2000 s at 10 mA/cm$^2_{geo}$	1 M H$_2$SO$_4$	*Energy Environ. Sci.* **2017**, 10, 2432
**Ru@IrO$_x$**	24 h at a constant anode voltage of 1.55 V$_{RHE}$	0.05 M H$_2$SO$_4$	*Chem* **2019**,5, 445–459
**Co-RuIr**	25 hours at 10 mA/cm$^2_{geo}$	0.1 M HClO$_4$	*Adv. Mater.* **2019**, 31, 1900510
**Ir-NiCo$_2$O$_4$**	70 hours at 10 mA/cm$^2_{geo}$	0.5 M H$_2$SO$_4$	*J. Am. Chem. Soc.* **2020**, 142, 18378
**Ir-Ag NTS**	6 hours at 5 mA/cm$^2_{geo}$	0.5 M H$_2$SO$_4$	*Nano Energy* **2019**, 56, 330
**Ir/GF**	10 hours at 10 mA/cm$^2_{geo}$	0.5 M H$_2$SO$_4$	*Nano Energy* **2017**, 40, 27
**Ir WNWs**	25000 s at 5 mA/cm$^2_{geo}$	0.1 M HClO$_4$	*Nanoscale*, **2018**, 10, 1892
**Ba$_2$YIrO$_6$**	1 hours at 10 mA/cm$^2_{geo}$	0.1 M HClO$_4$	*Nat. Commun.* **2016**, 7, 12363
**IrW/C**	8 hours at 5 mA/cm$^2_{geo}$	0.1 M HClO$_4$	*ACS Cent. Sci.* **2018**, 4, 1244
**IrNi NCs**	2 hours at 5 mA/cm$^2_{geo}$	0.1 M HClO$_4$	*Adv. Funct. Mater.* **2017**, *27, 1700886*

Table S8. Adsorption energy of adsorbates (*OH, *O and *OOH) on Ir_4O_8/V_2O_5 and bulk IrO_2 (110).

	Ir_4O_8/V_2O_5	IrO_2 (110)
ΔE_{OH*} (eV)	0.27	-0.24
ΔE_{O*} (eV)	2.02	1.35
ΔE_{OOH*} (eV)	3.29	2.82

Table S9. Comparison of water splitting performance with various recently reported state-of-the-art catalysts in all-pH media.

Electrocatalysts	$E_{@10\ mA\ cm^{-2}}$ / V	Current collector	Electrolyte	References
**IrO_2/V_2O_5 (±)**	1.50	Carbon fiber paper	0.5 M H_2SO_4	This work
	1.65		1 M PBS	
	1.49		1 M KOH	
**Ir-SA@Fe@NCNT**	1.51	Glassy carbon	0.5 M H_2SO_4	*Nano Lett.* **2020**, *20, 2120*
**Ir@GF**	1.55	Graphite foam	0.5 M H_2SO_4	*Nano Energy* **2017**,40 27
**IrAg**	1.55	Glassy carbon	0.5 M H_2SO_4	*Nano Energy* **2019**, 56, 330
**$MoSe_2$/NS/MoO_2 NB**	1.63	-	0.5 M H_2SO_4	*Nanoscale*, **2018**, 10, 9268
**$IrNi_{0.57}Fe_{0.82}$**	1.64	Carbon cloths	0.5 M $HClO_4$	*J. Mater. Chem. A*, **2017**, 5, 24836
**RuCu NS/C**	1.50	Glassy carbon	0.05 M H_2SO_4	*Angew. Chem. Int. Ed.* **2019**, 58, 13983
**RuTe**	1.52	Glassy carbon	0.5 M H_2SO_4	*Nat. Commun.* **2019**, 10, 5692
**$Ni_{0.1}Co_{0.9}P$**	1.89	Carbon fiber paper	1 M PBS	*Angew.Chem. Int.Ed.* **2018**, 57,15445
**Ni_3N@Ni-Bi NS/Ti**	1.95	Ti mesh	0.5 M K–Bi	*J. Mater. Chem. A*, **2017**, 5,7806
**S-$NiFe_2O_4$/NF**	1.95	Nickel foam	1 M PBS	*Nano Energy* **2017**, 40, 264
**Mn-Co-Bi/CC**	1.97	Carbon cloths	0.5 M K–Bi	*: J. Mater. Chem. A*, **2017**, 5, 12091
**CoO/$CoSe_2$**	2.18	Ti mesh	0.5 M PBS	*Adv. Sci.* **2016**, 3, 1500426
**Li-$IrSe_2$**	1.50	Carbon fiber paper	1 M PBS	*Angew. Chem. Int. Ed.* **2019**, 58, 14764
**Mo-Co_9S_8@C**	1.90	Carbon cloths	0.5 M Na_2SO_4	*Adv. Energy Mater.* **2018**, 8, 1801912
**NC/CuCo/$CuCoO_x$**	1.53	Nickel foam	1 M KOH	*Adv. Funct. Mater.* **2018**, 28, 1704447
**Am $FePO_4$/NF**	1.54	Nickel foam	1 M KOH	*Adv. Mater.* **2017**, 29, 1704574
**Ru_2Ni_2 SNs/NC**	1.58	Carbon fiber paper	1 M KOH	*Nano Energy* **2018**, 47, 1
**Ir_1@Co/NC**	1.60	Carbon fiber paper	1 M KOH	*Angew. Chem. Int. Ed.* **2019**, 131, 11994
**Co/β-Mo_2C@N-CNT**	1.64	Nickel foam	1 M KOH	*Angew. Chem. Int. Ed.* **2019**, 58, 4923

**N-NiMoO$_4$/NiS$_2$**	1.60	Carbon fiber paper	1 м KOH	*Adv. Funct. Mater.* **2019**, 29, 1805298
**Ni–Co-1T MoS$_2$**	1.48	Carbon fiber paper	1 м KOH	*Nat. Commun.*, **2017**, 8, 15377.

Table S10. Free energy correction values for adsorbates (*OH, *O and *OOH).

	E_ZPE (eV)	TS (eV)	E_H (eV)	a (eV)
*	-	-	-	-
*OH	0.362	0.0838	0.0482	0.326
*O	0.0735	0.0566	0.0323	0.0340
*OOH	0.4145	0.1437	0.0731	0.344

a : E_ZPE–TS+E_H
